# Built-in thiol mesoporous carbon immunosensor to detect carcinoembryonic antigen in human serum

**DOI:** 10.1007/s00604-026-07987-8

**Published:** 2026-04-13

**Authors:** Danilo Echeverri, Jennifer Laverde, Luis Gerónimo Restrepo, Nestor Llinás-Quintero, Diana López, Jahir Orozco

**Affiliations:** 1 Max Planck Tandem Group in Nanobioengineering, Institute of Chemistry, Faculty of Natural and Exact Sciences, University of Antioquia, Complejo Ruta N, Calle 67 N° 52–20, Medellín, 050010 Colombia; 2 Grupo Química Básica, Aplicada y Ambiente-Alquimia, Facultad de Ciencias Exactas y Aplicadas, Instituto Tecnológico Metropolitano de Medellín-ITM, Carrera 31 # 54-10, Medellín, 050034 Colombia; 3 Clinical Oncology Group, Colombian Cancer Foundation, Clinica Vida, Avenida 33 #63A- 18, Medellín, 050030 Colombia; 4 Química de Recursos Energéticos y Medio Ambiente, Institute of Chemistry, Faculty of Natural and Exact Sciences, University of Antioquia, Calle 70 No. 52-21, Medellín, 050010 Colombia

**Keywords:** Mesoporous carbon, Carcinoembryonic antigen, Immunosensor, Electrochemical detection, Differential pulse voltammetry, Colorectal cancer

## Abstract

A label-free electrochemical immunosensor for carcinoembryonic antigens (CEA) detection is reported based on a built-in thiol-mesoporous carbon architecture. The material was functionalized with 3-maleimidepropionic acid to immobilize anti-CEA antibodies. The immunosensor was characterized using field-emission scanning electron microscopy, X-ray photoelectron spectroscopy, cyclic voltammetry, and electrochemical impedance spectroscopy. Biorecognition events were monitored by differential pulse voltammetry, with current intensity dependent on protein concentration. The device exhibited a linear range of 1–10 ng mL^-1^ for CEA, with a clinically relevant limit of detection of 1.02 ng mL^-1^. The immunosensor successfully detected CEA in human serum samples, with results comparable to those obtained by a standard spectrophotometric method. This platform has proven to be a promising alternative for the prognosis and management of patients with colorectal cancer, as well as having significant potential for point-of-care diagnosis.

**Graphical Abstract:**

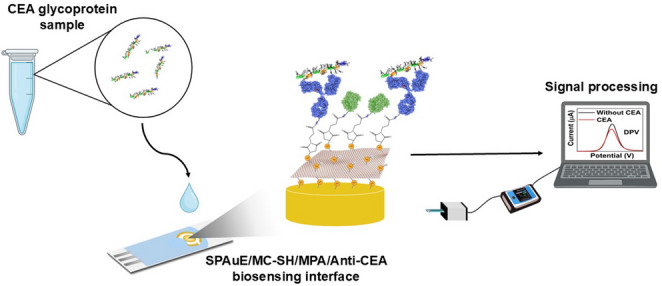

**Supplementary Information:**

The online version contains supplementary material available at 10.1007/s00604-026-07987-8.

## Introduction

Carbon-based mesoporous materials have attracted considerable interest for their ability to form complex nanoarchitectures with high structural control through relatively simple synthetic routes. These materials offer the advantage of tunable surface chemistry, enabling conjugation with diverse chemical species and biomolecules. Additionally, their large surface area and porosity support the development of multifunctional platforms with customizable interfacial properties [1]. Among carbonaceous materials, CMK-3-type mesoporous carbons have been used in biosensing, catalysis, and energy storage and conversion applications [2]. CMK-3-type materials are hierarchically ordered carbons with a 2D hexagonal mesoporous structure synthesized using silica templates. The linear arrangement of pores in CMK-3 enables the incorporation of elemental sulfur within its structure, effectively confining it and imparting electronic conductivity as the sulfur remains dispersed throughout the carbonaceous matrix [1]. This feature endows the material with significant potential in biosensor development [3].

Electrochemical immunosensors detect antigen-antibody interactions by forming a stable molecular immunocomplex at the electrode surface [4], where effective antibody immobilization is essential for the device’s analytical performance [5]. Immunosensors based on carbonaceous materials have been extensively reported [6]. However, those employing CMK-3-type carbons remain limited [1, 7]. On the other hand, bioreceptors can be immobilized on carbonaceous material-modified electrode surfaces using either physical or chemical methods [8]. Other approaches include crosslinking, i.e., coupling via free thiols with maleimides through a Michael addition reaction [5]. For example, 3-maleimidepropionic acid (MPA) has been used as a linker to immobilize biomolecules on glucose [9], enzymatic [10], and DNA [11] sensors via this route. However, the MPA linker has not been widely explored for the development of electrochemical immunosensors [12].

Conventional approaches for introducing thiol groups onto electrode surfaces usually involve surface grafting techniques, such as silanization of metal oxides [9], Aryl diazonium chemistry on carbon electrodes [13], covalent coupling with thiol-containing molecules (e.g., cysteamine) [14], thiolated polymeric coatings [15], or formation of simple or mixed thiolated self-assembled monolayers on metallic surfaces, where thiol moieties bind the electrode, and other functional groups remain exposed [16]. These methods effectively generate thiol groups at the electrode’s outermost surface, thereby restricting the density and spatial distribution of active sites to the external interface. In contrast, our work utilizes SPAuEs that are straightforwardly decorated with MC-SH, which provides abundant thiol anchoring sites for antibody immobilization on a stable platform, thereby integrating the high surface area and conductive properties of the mesoporous carbon framework. Unlike conventional silanization strategies, which often yield heterogeneous functional layers, our approach provides a more uniform distribution of active sites compared with other surface modification methods (e.g., electrochemical deposition), which may require multistep, time-consuming chemistries. Chemically linking the MC-SH to the SPAuEs provides stability to the platform by avoiding its leakage during the washing steps. To the best of our knowledge, this is the first report of an electrochemical immunosensor based on a CMK-3-type mesoporous carbon material with thiol groups intrinsically incorporated into the carbon framework, rather than introduced by surface-grafting techniques. Indeed, this material configuration has not been previously explored for the development of electrochemical immunosensors, underscoring the novelty of our platform. Moreover, bioreceptors linked to carbonaceous material via thiol groups could simplify and accelerate biosensing assembly.

Carcinoembryonic antigen (CEA) is a glycoprotein produced by normal human colonic epithelial cells and has been reported to be upregulated in tumorigenic and colonic adenocarcinoma cell lines [17]. Although its low clinical sensitivity limits its use as a screening biomarker, CEA has been approved by the Food and Drug Administration (FDA) for cancer prognosis and management. CEA is currently determined by Enzyme-Linked Immunosorbent Assay (ELISA), radioimmunoassay, chemiluminescent immunoassay [18], and proteomic approaches that combine liquid chromatography with mass spectrometry and data analysis using bioinformatic tools [19]. However, these methods are time-consuming, require centralized and advanced laboratory equipment, and rely on skilled personnel. Therefore, developing sensitive, specific, simple, rapid, and precise electrochemical immunosensors for detecting CEA in healthy and diseased conditions is needed. It would reduce analysis time and costs and enable disease diagnosis in resource-limited regions, particularly in low- and middle-income areas. In this context, various sensing strategies for CEA detection have been reported, including electrochemical, chemiluminescent, and fluorescence-based methods [20]. Among them, electrochemical platforms are particularly attractive due to their sensitivity, simplicity, and suitability for miniaturized and point-of-care applications.

**Fig. 1:**
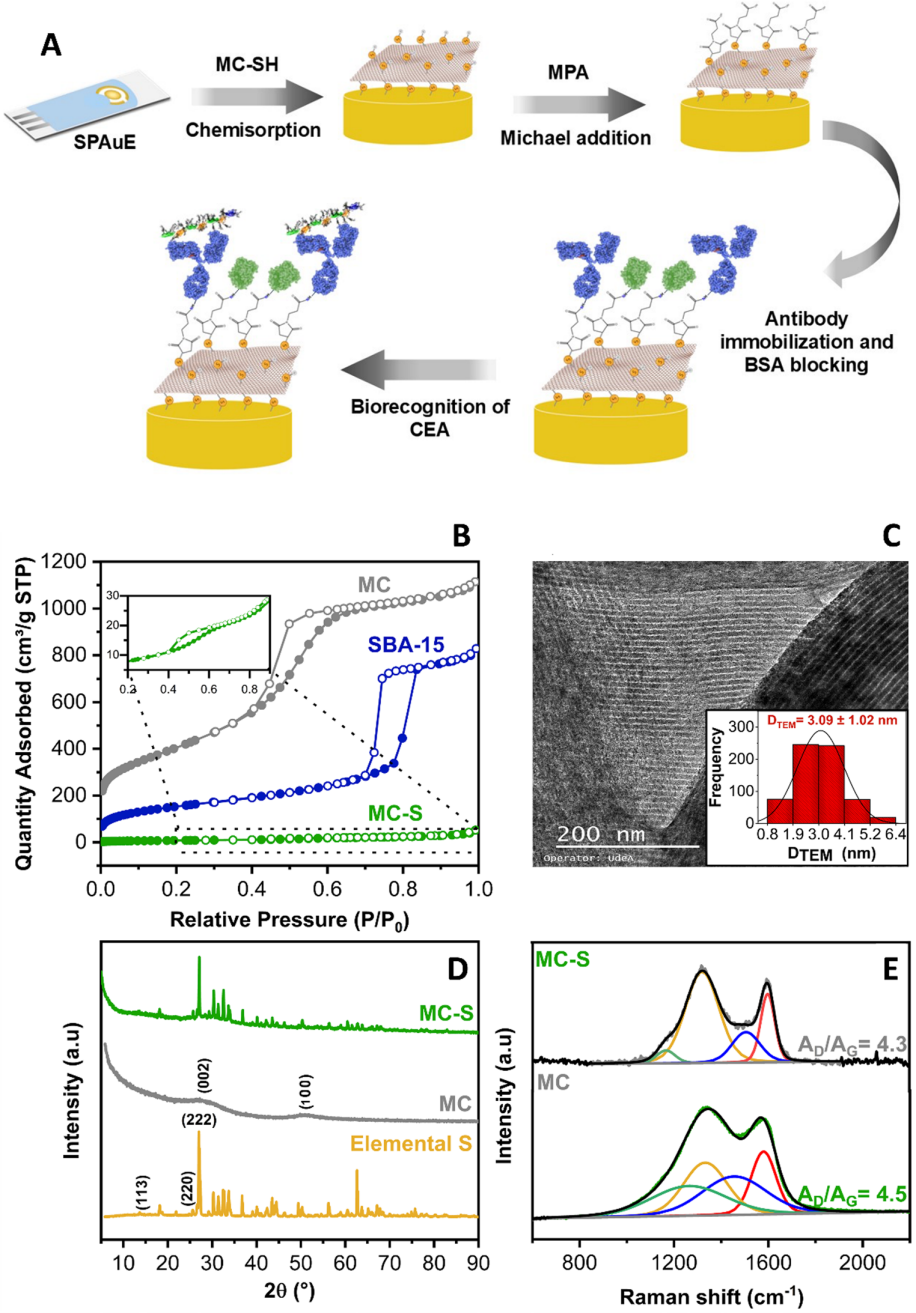
**A**) Step-by-step assembly of the electrochemical immunosensor. (1) MC-SH chemisorption. (2) MPA functionalization. (3) Antibody covalent immobilization and BSA blocking. (4) CEA glycoprotein biorecognition. Characterization of carbonaceous material. **B**) N_2_ adsorption/desorption isotherms for the materials. **C**) HRTEM micrograph and CMK-3-type mesoporous carbon (MC) pore size distribution. **D**) XRD patterns of elemental sulfur, MC, and MC-S composite. **E**) Raman spectra of the carbonaceous material before (MC) and after sulfur incorporation (MC-S)

This work incorporated thiolated mesoporous carbon (MC-SH) into screen-printed gold electrodes (SPAuEs) and functionalization with MPA to develop a label-free electrochemical immunosensor for CEA detection. MC-SH preparation involves a template-assisted synthesis process to obtain a CMK-3-type mesoporous carbon, sulfur incorporation into the pore structure, and chemical reduction to form thiol groups. The SPAuEs were modified with MC-SH via drop-casting and subsequently functionalized with MPA for covalent immobilization of the anti-CEA antibody. The conjugation strategy using MPA exploits the maleimide group, which reacts with surface-exposed thiol groups via a Michael addition. This reaction leaves carboxylic acid groups exposed, which can subsequently be activated with carbodiimide chemistry to enable covalent conjugation of the antibodies. Lacking a direct comparison with conventional thiol-modification strategies, this approach offers a suitable alternative when a thiolated mesoporous carbon layer serves as the functional interface on gold electrodes, ensuring stable linkage while preserving antibody activity. In contrast to conventional gold–thiol systems such as self-assembled monolayers, where thiol groups mainly provide single-point attachment to the electrode surface, the thiol functionalities in the built-in thiol mesoporous carbon are intrinsically distributed throughout the carbon framework. It enables dual functionality, providing both stable anchoring to the gold substrate and antibody coupling at multiple sites within a high-surface-area architecture. The step-by-step engineering of the electrochemical immunosensor on SPAuEs for CEA glycoprotein detection is illustrated in Fig. 1A. The physicochemical and electrochemical properties of the immunosensing platform were investigated in depth. The molecular biorecognition event was quantified by monitoring changes in the current generated from the oxidation of the ferrocyanide redox probe at the immunosensing interface, using differential pulse voltammetry (DPV) as a signal transduction technique. The immunosensor successfully detected CEA in serum samples from colorectal cancer patients. It provides a short-term assay with sensitive, label-free detection, demonstrating the feasibility of MC-SH as a biosensor platform and its potential for further analytical applications.

## Materials and methods

The supporting information (SI) section describes the reagents and solutions, apparatus, nanocomposite preparation and functionalization, physicochemical and electrochemical characterization, and assembly of the biosensing interface.

## Results and discussion

### Preparation and characterization of carbonaceous material

The SI section describes the nanocomposite preparation and functionalization, and nitrogen adsorption/desorption analysis was used to determine its textural properties and confirm the successful synthesis of a mesoporous carbonaceous material. Figure 1B presents the isotherms obtained for the silica template (SBA-15), the carbonaceous material before sulfur incorporation (MC), and the material after sulfur incorporation (MC-S). The SBA-15 template exhibits a type IV isotherm with an H1-type hysteresis loop in the relative pressure range of 0.4–0.8, indicative of capillary nitrogen condensation within the cylindrical mesopores. The MC material also displays a type IV isotherm, successfully replicating the mesoporous SBA-15 silica and abundant mesopores. The high surface area suggests a favorable distribution of active sites for hosting biomolecules and efficient dispersion of elemental sulfur [21]. As shown in **Table ****S1**, the MC material possesses a high surface area (1462.2 m^2^ g^− 1^), a pore volume of 1.7 cm^3^ g^− 1^, and an average pore size of 4.7 nm, confirming its mesoporous nature derived from the SBA-15 template. Upon the formation of the MC-S composite, a change in the adsorption/desorption isotherm is observed (Fig. 1B**)**. The incorporation of sulfur within the carbonaceous material’s pores significantly reduces nitrogen adsorption, likely due to pore blockage (**Table ****S1**, pore volume decreased to 0.1 cm^3^ g^− 1^). However, the persistence of the hysteresis loop in the P/P_0_ range of 0.4–0.8 indicates that the pores are not entirely blocked, and the material’s porous structure is largely retained after sulfur incorporation. Thermogravimetric analysis (TGA) under an inert atmosphere (**Fig. ****S1****A**) revealed a 68.2 wt%. sulfur content in the MC material, with the weight loss between 150 and 450 °C corresponding to sulfur sublimation. High-resolution transmission electron microscopy (HRTEM) (Fig. 1C) further confirmed the mesoporous structure of the MC material, revealing a pore size distribution centered at 3.09 ± 1.02 nm (*n* = 659), which falls within the mesopore range [22].

X-ray diffraction analysis (XRD) confirmed sulfur infiltration within the MC pores. Figure 1D shows the XRD patterns for the elemental sulfur, the MC material, and the MC-S composite. The XRD pattern of elemental sulfur presents sharp peaks corresponding to the crystalline orthorhombic phase with the space group fddd (ICSD 98-041-2326). The MC material shows a mainly amorphous structure with two broad peaks at 2θ = 30° and 51°, corresponding to the (002) and (100) planes of the ordered hexagonal structure, respectively (ICSD 98-061-7290). The XRD pattern of the MC-S composite exhibits peaks corresponding to the orthorhombic phase of sulfur. The MC-S diffractogram retains the amorphous character 20–35º region, attributed to the carbonaceous material. While the sulfur diffraction peak intensity is reduced due to pore confinement, its presence confirms that some sulfur remains on the surface. The MC-S synthesis aimed to incorporate sulfur into the pores and achieve uniform distribution, facilitating the dispersion of -SH functionalities formed during the reduction step and providing a homogeneous distribution of active sites. Scanning electron microscopy and energy-dispersive spectroscopy (SEM-EDX) confirmed the homogeneous sulfur distribution. **Fig. ****S1****B**. shows micrographs and elemental mappings for MC and MC-S materials. The MC material exhibits the characteristic ¨sausage-type¨ particle morphology, with lengths ranging from 0.1 to 0.3 μm. This morphology is retained after sulfur incorporation, and the sulfur elemental mapping confirms its homogeneous distribution.

Graphitization, a measure of order within the carbonaceous structure, directly influences electronic conductivity. A higher degree of graphitization, reflecting a greater population of aromatic rings, correlates with increased conductivity. Therefore, the MC-S composite must retain the conductive properties of the carbonaceous material. As shown in Fig. 1E, two peaks are observed at 1350 cm^− 1^ (D band) and 1580 cm^− 1^ (G band), corresponding, respectively, to the degree of structural disorder and the energy of sp2-type bonds (C = C bonds) from aromatic rings. When carbonaceous materials exhibit some disorder, band D usually overlaps with three bands at 1250 cm^− 1^, 1330 cm^− 1^, and 1450 cm^− 1^, corresponding to bands D4, D1, and D3, respectively. These bands are usually related to the presence of aromatic rings with at least six members (D1), with C-H and/or C-C type aliphatic groups linked to aromatic rings (D4), and with small aromatic rings of 3–5 members (D3) [23]. The band area ratio (AD/AG) was calculated using the D band area, defined as the sum of the D1, D3, and D4 contributions, to verify that the inclusion of sulfur did not alter the graphitization degree. The area ratios for MC and MC-S were 4.3 and 4.5, respectively (Table S1), indicating no significant change in the graphitization degree of the structure upon sulfur incorporation. The above relates to sulfur being incorporated mainly into the pores without significantly altering the chemical structure of the carbonaceous material.

Infrared spectroscopy analyzed the chemical structure of MC and MC-S (**Fig. ****S1****C**). The carbonaceous material before sulfur incorporation exhibits characteristic signals for the C = C (1565 cm^− 1^) and CH_3_ (1200 cm^− 1^) functional groups. The presence of oxygen is evident in the structure with C = O (1630 cm^− 1^), C-O (1139 cm^− 1^), and C-O-C (1055 cm^− 1^) type bonds that are commonly present in this type of carbonaceous material. After sulfur incorporation, the MC-S material presents signals at 1280 cm^− 1^ and 880 cm^− 1^, characteristic of elemental sulfur, as well as signals corresponding to the vibration of the C-S (1380 cm^− 1^ and 1320 cm^− 1^) and SO_3_ (1054 cm^− 1^) bonds. These latter signals suggest the formation of weak sulfur-oxygen bonds with the carbonaceous material [24].

XPS analysis (**Fig. ****S2**) corroborated the infrared spectroscopy results. The survey spectra (**Fig. ****S2****A**) show the presence of carbon and oxygen for the MC material, while elemental sulfur is evident for the MC-S composite. The total carbon content remained relatively unchanged after incorporating sulfur (91.9 and 91.6 at% for MC and MC-S, respectively). However, the oxygen content decreased from 8.1 at% in MC to 6.2 at% in MC-S, likely due to the formation of sulfur species with the carbonaceous matrix, consistent with the C-S functionalities observed in the infrared spectra. The XPS analysis detected 2.2 at% sulfur on the surface of the MC-S material. The high-resolution C1s spectra for the MC and MC-S material (**Fig. ****S2****B-C**) ​​present the four characteristic signals for the functionalities of carbonaceous materials. Signals corresponding to the C = C, C-C, and/or C-H, C-O, and C = O and/or COOH/COOR bonds are evident at 284.8, 285.5, 286.8, and 289.6 eV, respectively [25]. These signals are also present in the MC-S material, with no significant shifts, consistent with the infrared spectroscopy results. Sulfur incorporation results in a decrease in C-O bond content from 17.2 to 10.1 at%, likely due to the formation of C-S bonds, as evidenced by the infrared spectra. High-resolution O1s spectra (**Fig ****S2****D-E**) show three characteristic signals for C = O (530.7 eV), C-O (532.4 eV), and C-OH (533.8 eV) bonds [26]. Consistent with the C1s analysis, the atomic extent of C-O species decreases from 40.1 to 28.9 at%. The high-resolution S2p spectrum (**Fig. ****S2****F**) reveals three signals: two corresponding to elemental sulfur (Sp_3/2_ at 164.1 eV and Sp_1/2_ at 165.3 eV) and one indicating oxidized sulfur or C-S bonds (169.3 eV) [27]. Elemental sulfur constitutes 72.9 at% of the total surface sulfur, the dominant species compared to oxidized sulfur or C-S, suggesting its potential for reduction to -SH species.

### Biosensing interface assembly and characterization

**Fig. 2:**
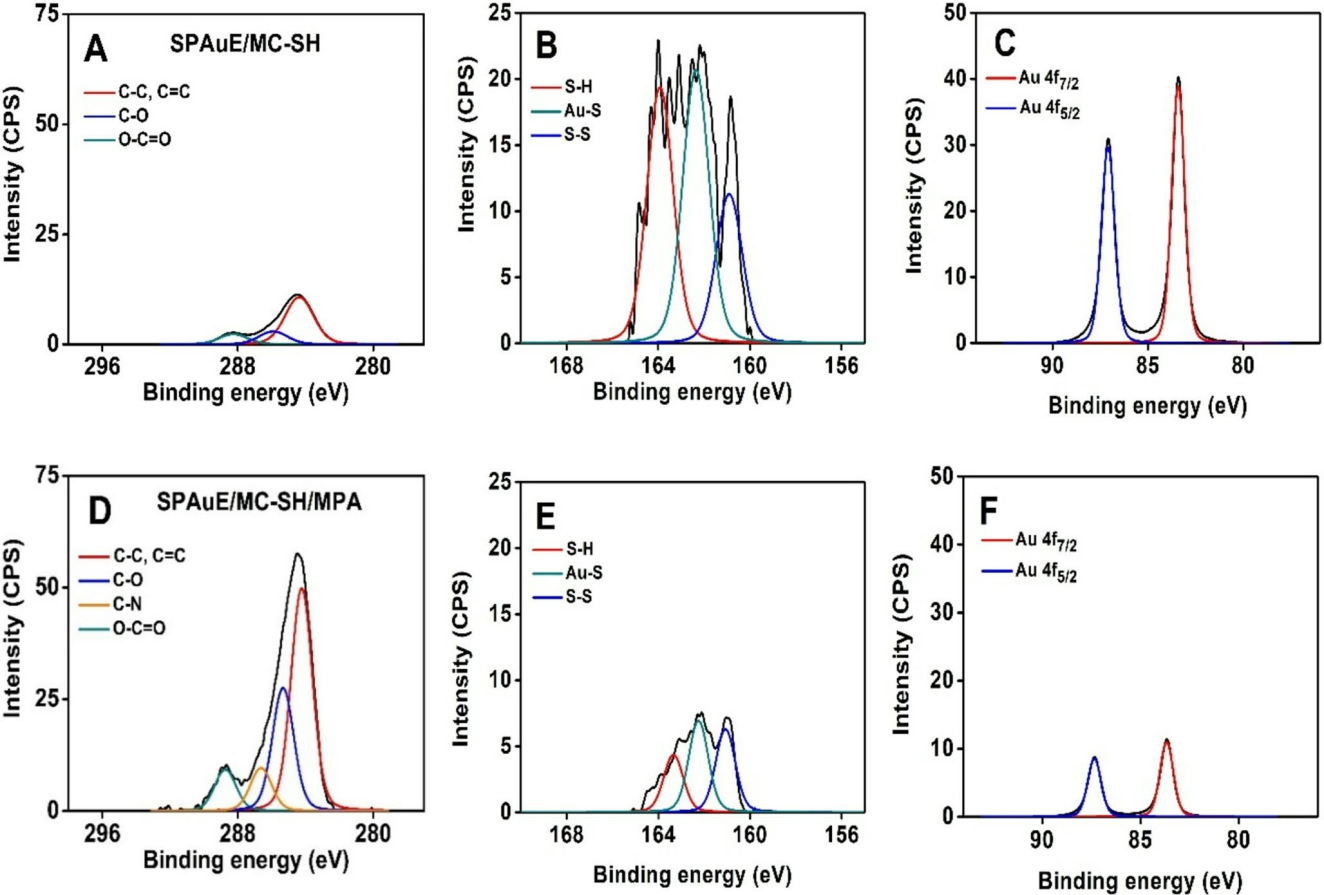
XPS core-level spectra of the MC-SH/SPAuE interface: **A**) C1s, **B**) S2p, **C**) Au4f. XPS core-level spectra of the MPA/MC-SH/SPAuE interface: **D**) C1s, **E**) S2p, **F**) Au4f

The screen-printed gold electrodes (SPAuEs) were modified with MC-SH by the chemisorption of sulfhydryl groups onto the gold surface. The thiol–gold interaction provides stable anchoring of the carbon layer, while the thiol functionalities distributed throughout the mesoporous framework enable antibody coupling within a high-surface-area architecture. In contrast to self-assembled monolayers, where thiol groups primarily serve as single-point anchors, the built-in thiol groups in MC-SH allow the carbon layer itself to act as the immobilization interface, supporting the proof-of-concept biosensing performance this study. The modified electrodes were characterized by FE-SEM coupled with EDX and XPS. **Fig. ****S4** and **Table ****S2** show the FE-SEM micrograph, the corresponding EDX spectrum, and the elemental composition of the SPAuE/MC-SH interface, respectively. The micrograph and EDX spectrum reveal a uniform distribution of the MC-SH on the gold electrode surface, suggesting strong chemisorption facilitated by the thiol groups. Figure 2A shows the high-resolution C1s spectrum, highlighting a peak at 284.4 eV attributed to graphite-like sp^2^-hybridized carbon, a second peak at 285.9 eV corresponding to sp^3^-hybridized carbon, and a peak at 288.3 eV attributed to the carboxyl group [28]. Furthermore, Fig. 2B presents the high-resolution spectrum of S2p, highlighting two peaks at 162.4 and 163.9 eV [29], attributed to gold-bound thiols and free thiols, respectively [27], and a peak at 160.9 eV, attributed to polysulphides [29]. These results confirm that SPAuEs were successfully modified with the thiolated mesoporous carbon material.

The thiols exposed on the electrode surface were used to functionalize the SPAuE/MC-SH interface with carboxylic acids by reaction with 3-maleimide propionic acid (MPA). This reaction proceeds via a Michael addition of the thiol groups to the maleimide, yielding carboxylic acids on the electrode surface that are available for bioreceptor coupling. XPS was used to characterize the SPAuE/MC-SH/MPA interface to confirm this. Figure 2D shows the increase in carbon content after MPA conjugation, as indicated by the higher intensity of the peaks corresponding to sp² and sp³ hybridized carbon concerning the SPAuE/MC-SH interface. Furthermore, the component at 286.6 eV, corresponding to the C-N bond in the maleimide [31], was identified [30]. The peak associated with carboxylic acids showed increased intensity, while the peaks attributed to the sulfur (Fig. 2E) and gold components (Fig. 2C-F) showed decreased intensity. These results confirm the successful conjugation of MPA to the thiolated interface.

**Fig. 3:**
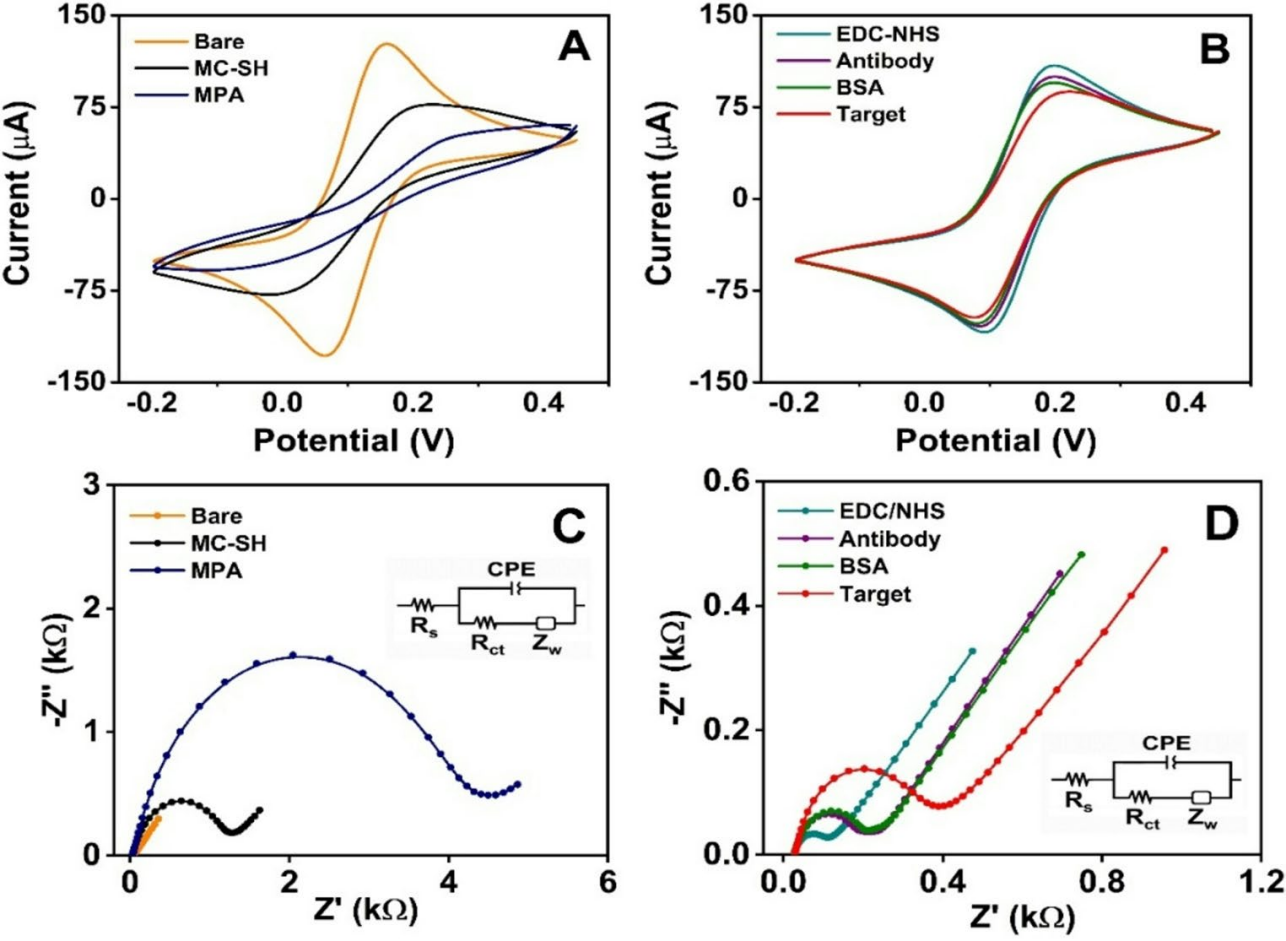
The cyclic voltammograms (**A** and **B**) and EIS-derived Nyquist plots (**C** and **D**) illustrate the electrochemical characterization at each stage of the immunosensor assembly, conducted in a PBS 1X (pH 7.4) solution containing 5 mM [Fe(CN)_6_]^4−/3−^ as the redox probe. The steps shown are bare SPAuE (orange), MC-SH/SPAuE (black), MPA/MC-SH/SPAuE (blue), EDC/NHS/MPA/MC-SH/SPAuE (cyan), anti-CEA/MPA/MC-SH/SPAuE (purple), BSA/anti-CEA/MPA/MC-SH/SPAuE (green), and CEA/BSA/anti-CEA/MPA/MC-SH/SPAuE (red). The insets in **C** and **D** depict the equivalent circuit used to fit the impedance spectra

Figure 3 presents the electrochemical characterization by CV and EIS of each step in the immunosensor assembly using the 5 mM [Fe(CN)_6_]^4−/3−^ redox probe in PBS 1X, pH 7.4, as the supporting electrolyte. Electrochemical characterization by CV for each SPAuE modification step was assessed by monitoring changes in the electroactive area (*A*_*e*_), calculated using the Randles–Sevcik equation. Figure 3A and **B** show the CVs corresponding to a quasi-reversible electrochemical reaction. The sequential decrease in *A*_*e*_ of the SPAuE/MC-SH interface, and after MPA conjugation, compared to the bare electrode, was attributed to the electrostatic repulsion between the carboxylic acids on the surface and the negatively charged redox probe, which decreased the electron transport kinetics. On the other hand, the *A*_*e*_ increased after the activation of carboxylic acids with EDC/NHS. This result was attributed to the lower electrostatic repulsion between the redox probe and the electrode interface, as the N-hydroxysuccinimide ester is uncharged and facilitates electron transfer. In contrast, an anti-CEA antibody covalently bound to the SPAuE/MC-SH/MPA interface, and BSA, which blocked the surface, hindered electron transfer from the redox probe by electrostatic repulsion and steric hindrance, decreasing the *A*_*e*_. Likewise, the antigen-antibody immune complex formation was confirmed by the decrease in the *A*_*e*_ attributed to the blockage of electron transfer by the biomolecules on the electrode surface. The results are summarized in **Table ****S3**.

In addition, EIS experiments characterized each step of the immunosensor assembly. Figure 3C and **D** show the Nyquist plot corresponding to each modification step. The interfacial electrical properties were modeled by the equivalent circuit *R*_*s*_ (*CPE* [*R*_*ct*_ *Z*_*w*_]), where *R*_*s*_ is the electrolytic solution resistance, *CPE* is the constant phase element, *R*_*ct*_ is the charge transfer resistance, and *Z*_*w*_ is Warburg’s impedance. The CPE describes the non-ideal capacitance, according to the equation *CPE* = − *1/(iωC)*^*n*^, where ω is the angular frequency, *C* is the capacitance, and *n* is the CPE exponent, which varies between 1.0 for a pure capacitor, 0.5 for a rough electrode, and 0 for a pure resistor [31]. The high-frequency region in the Nyquist plot corresponds to the charge-transfer process, while the low-frequency region corresponds to the diffusion of electroactive species. **Table ****S3** summarizes the resulting electrical parameters from fitting with the equivalent circuit. The increase in *R*_*ct*_ of the SPAuE/MC-SH interface (*R*_*ct*_ = 1198 ± 80 Ω) compared to the bare SPAuE (*R*_*ct*_ = 15.8 ± 2.3 Ω) indicates an efficient chemisorption of MC-SH onto the gold surface. The *R*_*ct*_ increased significantly after MPA conjugation (*R*_*ct*_ = 4392 ± 316 Ω), attributed to the electrostatic repulsion between the surface carboxylic acids and the negatively charged redox probe, confirming the successful MPA conjugation to the thiolated interface. In contrast, *R*_*ct*_ decreased to 86.7 ± 7.4 Ω after activation of carboxylic acids with EDC/NHS, attributed to the lower electrostatic repulsion [32]. The conjugation of the anti-CEA antibody and BSA to the N-hydroxysuccinimide esters increased the *R*_*ct*_ to 143.2 ± 8.9 and 184.3 ± 29.8 Ω, respectively. This increase is attributed to the biomolecules immobilized on the electrode surface, which hindered electron transfer from the redox probe. The rise in *R*_*ct*_ confirms the successful covalent immobilization of the bioreceptor and BSA on the SPAuE/MC-SH/MPA interface. The antigen-antibody immune complex significantly hindered electron transfer, resulting in an increase in *R*_*ct*_ to 324.8 ± 27.1 Ω. Regarding the fitted equivalent-circuit parameters, *R*_*s*_ remained nearly constant throughout all modification steps, confirming that the electrolyte solution resistance was not significantly affected during the surface assembly. At the same time, the CPE reflected the non-ideal capacitive behavior of the interface, progressively evolving as surface roughness, heterogeneity, and biomolecular coverage increased during functionalization and biorecognition. The exponent *n* value was close to 0.8 at all steps, reflecting non-ideal capacitive behavior associated with interfacial heterogeneity. These EIS results are consistent with those obtained from CV measurements and confirm the successful assembly of the electrochemical immunosensor. The increase in charge-transfer resistance observed after MC-SH modification reflects the formation of a nanostructured carbon layer that partially blocks direct access of the redox probe to the underlying gold surface, as evidenced by EIS. This effect is associated with increased interfacial resistance arising from the negatively charged, built-in thiolated nanostructured carbon interface, which repels the negatively charged redox probe in solution, rather than with the mesoporous carbon’s poor intrinsic conductivity. Importantly, DPV measurements recorded after full sensor assembly display clear and reproducible faradaic responses, confirming that effective electron transfer through the MC-SH layer is maintained and that the sensing mechanism relies on controlled modulation of interfacial electron transfer upon biorecognition.

### Optimization of the experimental parameters involved in the CEA detection and analytical performance

DPV was used as a transduction technique to monitor the molecular biorecognition event, owing to its high sensitivity, which minimized capacitive current and maximized the faradaic response. The decrease in DPV current intensity was attributed to the formation of immune complexes, which hindered electron transfer at the electrode/electrolyte interface. The molecular biorecognition event was monitored by the normalized change in DPV current intensity, calculated as the relative response $RR\%=\left[\right({I}_{Immunosensor}-{I}_{CEA})/{I}_{Immunosensor}]x100\%$, where ${I}_{Immunosensor}$ represents the DPV current intensity of the BSA/anti-CEA/MPA/MC-SH/SPAuE interface, and ${I}_{CEA}$ represents the DPV current intensity after binding to the CEA glycoprotein. It optimized two experimental parameters related to the antibody bioreceptor (antibody concentration and antigen-antibody interaction time) to maximize analytical performance. The maximum signal-to-noise ratio (S/N) required to detect 10 ng mL^− 1^ of CEA was used as the criterion for selecting the optimal values. First, it assessed antibody concentrations (50, 100, and 150 µg mL^− 1^) to optimize antibody density, maximize antigen-antibody immune complex formation, and minimize steric hindrance at the electrode surface. As shown in **Fig. ****S5****A**, the S/N ratio 1.9 was maximal at an antibody concentration of 100 µg mL^− 1^, chosen to optimize the antigen-antibody interaction time. The effect of the antigen-antibody interaction time on the immunosensor performance was evaluated over 30 to 60 min. **Fig. ****S5****B** shows that the maximum S/N ratio of 5.2 was achieved at 60 min, indicating efficient immunoreaction. Therefore, the optimal parameters for the antibody were a concentration of 100 µg mL⁻¹ and 60 min antigen-antibody interaction time.

**Fig. 4:**
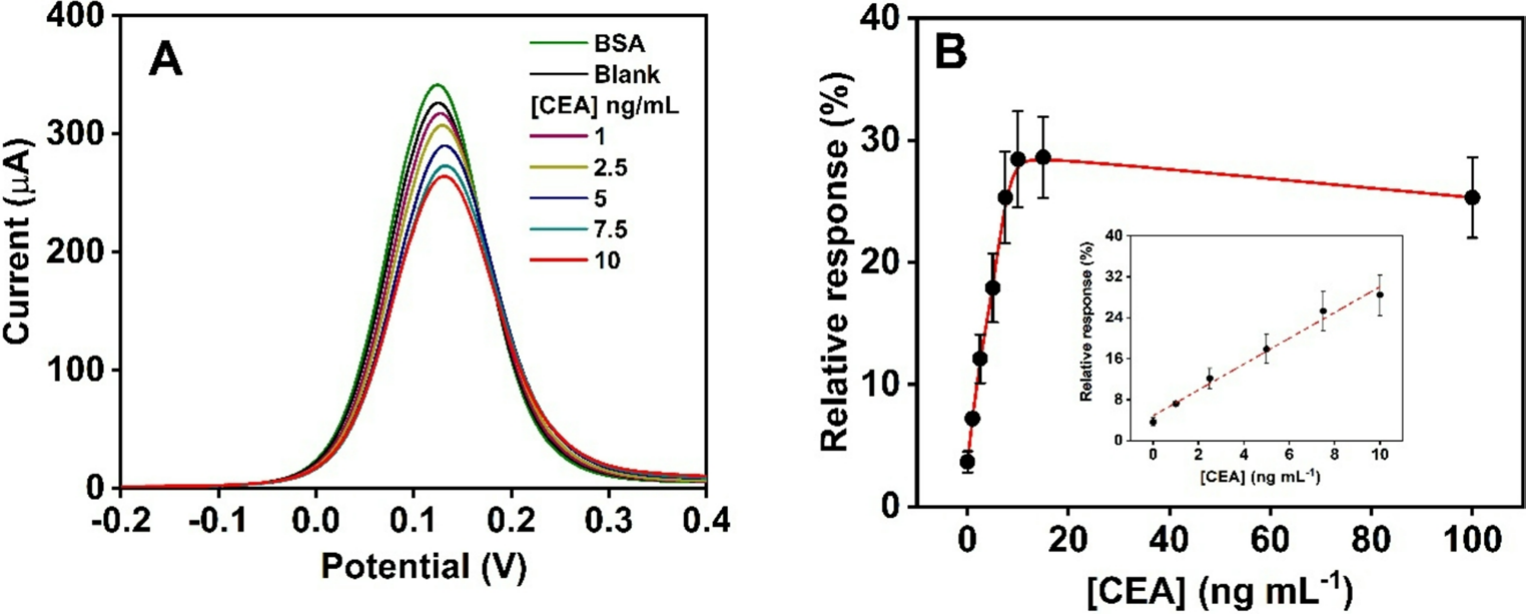
**A**) DPV response for different CEA glycoprotein concentrations, from 1 to 10 ng mL^− 1^. **B**) Resultant relative variation of DPV current with the CEA concentration. The inset shows the linear regression of relative response (RR%) versus CEA concentration, with the mean ± SD from triplicate measurements using three independent electrodes (*n* = 3)

We assessed the immunosensor analytical performance once optimal conditions for CEA glycoprotein detection were determined by analyzing different solutions containing known concentrations of CEA. Figure 4A shows the decrease in DPV current intensity with increasing CEA. As depicted in Fig. 4B, the immunosensor exhibited a dynamic linear range of 1–10 ng mL^-1^, as described by the linear regression equation *RR % = 2.522 [CEA] + 4.853*, with a correlation coefficient (*R*^*2*^) of 0.985. The calibration linear range was defined using linear least-squares fitting, selecting the concentration interval with the highest correlation and a proportional relationship between the normalized DPV response and the CEA concentration. Accordingly, a linear response was observed over 1–10 ng mL^-1^, with higher concentrations excluded due to deviations from linearity. Analytical parameters were calculated from the calibration curve. The LOD and LOQ were determined as 1.02 and 3.41 ng mL^-1^, respectively, using the 3σ and 10σ criteria. These values were calculated from the standard deviation obtained from ten independent blank measurements, which showed a signal variation of 3.67 ± 0.86%. The achieved sensitivity enables the detection of CEA concentrations within clinically relevant ranges, encompassing commonly reported prognostic cut-off values (e.g., 5 ng mL^-1^) for colorectal cancer, as well as stage-specific individualized thresholds of approximately 3.5–7.4 ng mL^-1^ [33, 34]. Therefore, the established dynamic range of 1–10 ng mL^-1^ adequately covers these clinically significant intervals. For concentrations above 10 ng mL^-1^, standard sample dilution procedures can be applied to bring the measurement within the validated linear range. While higher sensitivity is possible with complex amplification strategies, our goal is to demonstrate MC-SH as a simple, label-free platform to evaluate its suitability as a biosensing material, achieving sufficient performance to distinguish normal and elevated CEA levels. **Table ****S5** presents other electrochemical immunosensors for CEA detection based on screen-printed electrodes. Notably, the assay performed on SPAuE requires only a small sample volume and produces a signal within 60 min, enhancing the system’s reproducibility, speed, and portability.

### Biosensor specificity, reproducibility, repeatability, and stability

**Fig. 5:**
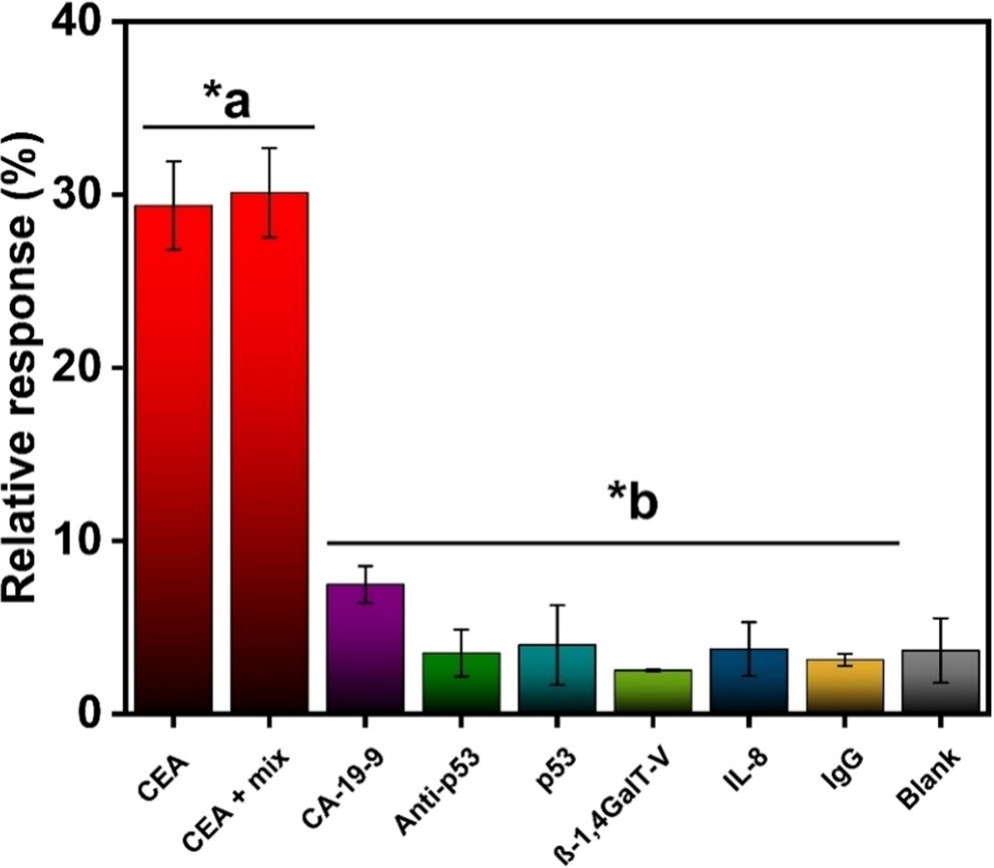
Relative signal variation for 10 ng mL^− 1^ of CEA in the presence of interferenets such as CA-19-9 glycoprotein, anti-p53 antibodies, p53 antigen, β-1,4-GalT-V glycoprotein, interleukin-8, and IgG antibodies. Relative response was calculated as $RR\%=\left[\right({I}_{Immunosensor}-{I}_{CEA})/{I}_{Immunosensor}]x100\%$, where ${I}_{Immunosensor}$ represents the DPV current intensity of the BSA/anti-CEA/MPA/MC-SH/SPAuE interface, and ${I}_{Sample}$ represents the DPV current intensity after sample incubation. *a, Significantly different from the blank sample (*p* < 0.05). *b, Significantly different regarding the CEA sample (*p* < 0.05)

The specificity of the immunosensor was evaluated by monitoring the normalized changes in DPV current intensity response to biomolecules with potential cross-reactivity to the anti-CEA antibody, including CA-19-9 and p53 antigens, anti-p53 and IgG antibodies, β-1,4-galactosyltransferase-V (β-1,4-GalT-V) glycoprotein, and chemokine interleukin 8 (IL-8). The CA-19-9 antigen was measured at 150 U mL^− 1^ from human serum, diluted two-fold in PBS. This level is typical in cancer patients. p53 antigen, anti-p53 and IgG antibodies, and β-1,4-GalT-V glycoprotein were measured at 1 µg mL^− 1^. The IL-8 chemokine was measured at 2 ng mL^− 1^. These concentrations were higher than the CEA concentration used to corroborate the assay’s reliability. Figure 5 shows the normalized change in DPV current intensity for the immunosensor in the presence of the six interferents evaluated individually and in a mix with CEA at 10 ng mL^− 1^. The results showed that the RR % was higher for the CEA glycoprotein (RR % = 29.4 ± 2.6) compared to CA-19-9 antigen (RR % = 7.5 ± 1.1), anti-p53 antibodies (RR % = 3.5 ± 1.4), p53 antigen (RR % = 4.0 ± 2.3), β-1,4-GalT-V glycoprotein (RR % = 2.5 ± 0.05), IL-8 chemokine (RR % = 3.8 ± 1.6), and IgG antibodies (RR % = 3.1 ± 0.4). The RR % shows a differential response, with significant differences observed by paired t-test and 1-way ANOVA at the 95% level of significance. These results indicate that the immunosensor was highly selective and specific for CEA glycoprotein detection. The device assembled up to the BSA blocking step on ten independent electrodes exhibited high inter-electrode reproducibility (RSD = 1.88%), as shown in **Fig. ****S6**. We assessed the intra-electrode repeatability and inter-electrode reproducibility of the immunosensor for target detection by measuring the DPV current response of 10 ng mL^− 1^ of CEA glycoprotein. The repeatability and reproducibility, expressed as relative standard deviation (RSD), were 6.7% (*n* = 3) and 8.5% (*n* = 5), respectively. These results indicate that the immunosensor exhibited acceptable reproducibility for detecting the CEA glycoprotein, but further optimization could improve its performance.

Furthermore, we measured the normalized change in DPV current intensity to detect 10 ng mL^− 1^ of CEA for one week to determine the stability of the immunosensor over time. Measurements used two electrodes stored in 1X PBS (pH 7.4) at 4 °C in a dark, humid chamber before electrochemical detection. The normalized change in DPV current intensity was recorded on the first day, followed by measurements one day later and every two days for seven days. As shown in Fig. S4B, the measurements’ mean and standard deviation (SD) from the first day were compared with those taken on each subsequent day. The initial RR % (24.4 ± 1.1) was the reference to assess stability, with three times the baseline standard deviation (3 SD) selected as the upper and lower control limits. After 1 day of storage, the immunosensor retained 93% of the initial RR%, with an RSD of 11.5%; after 5 days of storage, it maintained only 85% of its activity, with an RSD of 4.9%. The decrease in the immunosensor response was attributed to the loss of anti-CEA antibody activity and the passivation of the gold-working electrodes. Additionally, the decay in stability could be attributed to the retro-Michael reaction of the MPA-thiol conjugate in aqueous solution. The thioether bond formed via maleimide–thiol coupling undergoes slow retro-Michael and thiol exchange reactions in aqueous environments containing competing thiols [35]. However, these processes are time-dependent and are expected to be limited within the short incubation times used in this work (30 min). The immunosensor retains specific target recognition for up to 5 days after antibody immobilization and BSA blocking, indicating functional stability within the relevant operational timeframe. In addition, RR% signals remained within control limits, indicating that the platform worked reliably for up to 5 days and that the immunosensor is suitable for short-term use. While five days of stability may be acceptable for applications such as point-of-care diagnostics or rapid assays, it may be insufficient for long-term monitoring or for devices requiring extended shelf life. Therefore, a five-day lifespan may be considered a limitation in industrial or clinical settings, where (bio)sensors are expected to remain functional for weeks or even months. However, this stability is comparable to that of other antibody-based electrochemical immunosensors, which report lifetimes of 3–7 days under similar refrigerated conditions [36, 37]. Future work will focus on improving stability strategies, for example, by incorporating crosslinking agents, stabilizing additives, protective surface coatings, and inert atmosphere storage, or exploring other, more stable bioreceptors, to enhance robustness and extend the shelf-life of the device.

### Analysis of serum samples

Unlike recovery experiments with CEA in directly spiked serum samples, this proof-of-concept tested the immunosensing platform on real serum samples. Furthermore, matrix interference was assessed using the CA-19-9 antigen spiked into a human serum matrix (Abcam ab116024) and diluted two-fold in PBS to minimize matrix interference, reflecting typical clinical practice. At a clinically relevant concentration of 150 U mL^− 1^, the relative response for CEA (29.4 ± 2.6%) was markedly higher than that of CA-19-9 (7.5 ± 1.1%), demonstrating the high specificity and selectivity of the immunosensor in the serum matrix.

Six human serum samples (obtained from Clínica Vida, Medellín, Colombia, in accordance with the local ethical committee’s guidelines) with varying CEA levels were analyzed to demonstrate the practical application of the proposed electrochemical immunosensor. Before measurement, the samples were diluted two-fold in PBS. Subsequently, the proposed immunosensor detected CEA concentration in the samples based on the current-concentration relationship. The CEA glycoprotein concentration determined by a CEA ELISA kit was comparable with our electrochemical immunosensor (**Table ****S4**). CEA concentrations below the LOQ were considered not detected (ND) and were excluded from statistical analyses. Quantitative evaluation was performed exclusively using concentrations ≥ LOQ within the linear range. Furthermore, a paired t-test of the outcomes at the 95% confidence level (*p*-value = 0.75) revealed no statistically significant difference between the two methods. The Pearson correlation coefficient of *r* = 0.89 (*p* = 0.11, *n* = 4) between the ELISA and immunosensor results for serum samples from cancer patients (samples 3–6) indicated a strong positive correlation between the two methods. The lack of statistical significance is attributable to the limited sample size; however, the result nonetheless supports the agreement between the electrochemical assay and the conventional ELISA method. These results demonstrated the reliable performance of the immunosensor in detecting CEA in real samples. When cancer patients exhibit CEA concentrations above 10 ng mL^− 1^, simple sample dilution yields concentrations within the linear range. Slightly diluting samples is a common practice that doesn’t compromise accuracy and has been shown to reduce interferences [38–40]. In future work, the platform could be further optimized to expand the linear range or enhance sensitivity by adjusting antibody density, sensor surface area, or signal amplification strategies. Furthermore, antifouling strategies, such as polymer coatings, could be explored to enable direct application of undiluted serum onto the sensor surface, eliminating the need for sample dilution.

Although the electrochemical immunosensor presented here as a materials-level proof of concept, the platform’s performance is currently limited by sensitivity and long-term stability. Nevertheless, the electrochemical interface offers opportunities for future development, including multiplexed detection via selective functionalization with distinct recognition elements, potential integration with portable electrochemical readers, and single-use applications for biomarker monitoring. This immunosensor could serve as a practical detection tool in clinical or decentralized settings. Realizing these advanced applications will require optimization of the sensing interface, signal transduction strategy, and operational stability. Implementing the platform for probe-free or reagent-less detection represents a valuable future direction. Indeed, incorporating a redox probe into the built-in carbonaceous network could eliminate the need for redox probes in solution, simplifying the approach and enhancing the device’s portability. In addition, the highly porous mesoporous carbon provides a large electrochemically accessible surface area and well-defined interfacial capacitance, enabling probe-free signal transduction via electrochemical capacitance spectroscopy (ECS). Biomolecular recognition within the porous network may induce measurable changes in capacitance, potentially enhancing sensitivity while eliminating the need for redox probes.

## Conclusion

We synthesized a functional, built-in thiol-mesoporous carbon material deposited onto an SPAuE platform and functionalized with MPA for covalent immobilization of antibodies, and we explored it for the first time as a biosensing platform for CEA detection. The mesoporous carbon interface provided a large surface area and thiol groups for conjugation with MPA, which acts as a linker for the covalent immobilization of anti-CEA antibodies. This approach, previously unexplored in the development of immunosensors, offers a novel method for straightforward antibody immobilization on SPAuEs. Electrochemical and physicochemical characterization by XPS confirmed the successful assembly of the immunosensor platform. The high performance of the electrochemical immunosensor enabled the detection of CEA glycoprotein concentrations in human serum samples, demonstrating its potential to determine the serostatus of colorectal cancer patients. It is worth noting that the proposed MC-SH-based platform enables detection within 60 min and can be implemented on SPAuEs compatible with portable and handheld electrochemical devices. These features, combined with its simplicity and rapid response, underline the promise of this immunosensor for clinical applications in decentralized settings and support its potential as a point-of-care diagnostic tool.

## Supplementary Information

Below is the link to the electronic supplementary material.

Supplementary file1 (DOCX 6.38 MB)

## Data Availability

Data available upon request.
