# Supplementary material for: Built-in thiol mesoporous carbon immunosensor to detect carcinoembryonic antigen in human serum
Source: Mikrochim Acta. 2026 Apr 13;193(5):309. doi: 10.1007/s00604-026-07987-8 (PMC13070978; doi:10.1007/s00604-026-07987-8)
Supplement: Supplementary file 1 — Supplementary file1 (DOCX 6.38 MB) [file 604_2026_7987_MOESM1_ESM.docx]

**Built-in thiol mesoporous carbon immunosensor to detect carcinoembryonic antigen in human serum**

Danilo Echeverri^a,^, Jennifer Laverde^b,^, Luis Gerónimo Restrepo^c^, Nestor Llinás-Quintero^c^, Diana López^d^ and Jahir Orozco^a*^

*^a^Max Planck Tandem Group in Nanobioengineering, Institute of Chemistry, Faculty of Natural and Exact Sciences, University of Antioquia.*

*Complejo Ruta N, Calle 67 N° 52–20, Medellín 050010, Colombia*

*^b^Grupo de Química Básica, Aplicada y Ambiente-Alquimia, Facultad de Ciencias Exactas y Aplicadas, Instituto Tecnológico Metropolitano de Medellín-ITM, Carrera 31 # 54-10, Medellín 050034, Colombia*

*^c^Clinical Oncology Group, Colombian Cancer Foundation, Clinica Vida, Avenida 33 #63A-18, Medellín 050030, Colombia*

*^d^Química de Recursos Energéticos y Medio Ambiente, Institute of Chemistry, Faculty of Natural and Exact Sciences, University of Antioquia, Calle 70 No. 52-21, Medellín 050010, Colombia*

*Corresponding author*:*

[*grupo.tandemnanobioe@udea.edu.co*](mailto:grupo.tandemnanobioe@udea.edu.co)

**SUPPORTING INFORMATION**

**Experimental Procedures**

[**1.** **Materials** 2](#_Toc177822907)

[**2.** **Apparatus** 2](#_Toc177822908)

[**3.** **Sulfur-doped mesoporous carbon preparation** 3](#_Toc177822909)

[**4.** **MC-S functionalization with thiol groups** 5](#_Toc177822910)

[**5.** **SPAuE pretreatment** 5](#_Toc177822911)

[**6.** **Immunosensor preparation** 6](#_Toc177822912)

[**7.** **Electrochemical measurements** 7](#_Toc177822913)

[**8.** **Analytical performance** 7](#_Toc177822914)

[**9.** **Analysis of human serum samples** 7](#_Toc177822915)

[**References** 13](#_Toc177822916)

1. **Materials**

Silica mesoporous SBA-15, hydrofluoric acid (HF 48 % *v*/*v*), furfuryl alcohol, aluminum chloride (AlCl_3_), sodium cyanoborohydride (NaBH_3_CN reagent grade 95 %), carcinoembryonic antigen (CEA, ref. 219369), mouse monoclonal anti-CEA antibody (anti-CEA, ref. C2331), bovine serum albumin (BSA), 2-(N-morpholino) ethanesulfonic acid sodium salt (MES), N-(3-dimethylaminopropyl)-N'-ethylcarbodiimide hydrochloride (EDC) and N-hydroxysuccinimide (NHS) were purchased from Sigma-Aldrich (St. Louis, MO, USA). Sulfur (99.5 %) was purchased from Kanto Chemical (Tokyo, Japan). Potassium ferricyanide (K_3_[Fe(CN)_6_]), potassium hexacyanoferrate trihydrate (K_4_[Fe(CN)_6_].3H_2_O), and ethanol (CH_3_CH_2_OH reagent grade ≥ 99.9 %) were purchased from Merck Millipore (Darmstadt, Germany). Disodium hydrogen phosphate (Na_2_HPO_4_) and Carbon disulfide (CS_2_, 99.5 %) were acquired from PanReac AppliChem (Darmstadt, Germany). Potassium dihydrogen phosphate (KH_2_PO_4_), potassium chloride (KCl), and sodium chloride (NaCl) were obtained from J.T.Baker® (Xalostoc, Mexico). Sulfuric acid (H_2_SO_4_) was purchased from Honeywell Fluka^TM^ (Seelze, Germany). 3-Maleimidopropionic acid (MPA) was purchased from Santa Cruz Biotechnology (Dallas, TX, USA). Human CEA ELISA kit (ab264604), Human cancer antigen CA-19-9 (ab116024), recombinant human p53 protein (ab199593), recombinant β-1,4-Galactosyltransferase-V glycoprotein (β-1,4-GalT-V, ab160437), interleukin-8 (IL-8, ab48481), mouse monoclonal anti-p53 antibody (anti-p53, ab28), and anti-human IgG (anti-IgG, ab97161) were purchased from Abcam (Cambridge, MA, USA). All chemicals were used without any further treatment. All solutions were prepared using deionized water (18.2 MΩ cm) from a Thermo Scientific Barnstead GenPure ultrapure water purification system. Phosphate buffer saline (PBS 1X) consists of 0.01 M phosphate buffer, 0.138 M NaCl, and 0.0027 M KCl solutions with adjusted pH at 7.4.

1. **Apparatus**

Nitrogen adsorption/desorption isotherms (77 K, ASAP 2020) were obtained to confirm that the carbonaceous material replicated the porosity of the SBA-15 silica template and to verify the incorporation of sulfur into the material's pores. The results were analyzed using the Brunauer–Emmett–Teller (BET) method. The linear array of pores was observed by transmission electron microscopy (TEM; FEI Tecnai F20 Super Twin TEM/STEM) operated at 200 kV. The pore-size distribution from TEM analysis was determined using ImageJ. The crystalline phases of the carbonaceous material, elemental sulfur, and the sulfur/carbon composite were evaluated by X-ray powder diffraction (XRD) using an X-ray diffractometer (Panalytical, Empyrean Serie 2, Malvern, UK). The diffractograms were collected over the 5-90° range with a step size of 0.01° using Co Kα radiation. Phase identification was performed using the Inorganic Crystal Structure Database (ICSD, Bonn, Germany). The sulfur content incorporated into the MC-S material was determined by thermogravimetric analysis (TGA, SDT-Q600, TA Instruments) under an inert atmosphere from room temperature to 600 °C at a heating rate of 5 °C min^-1^. The graphitic properties of the carbonaceous material before and after sulfur incorporation were studied by Raman spectroscopy (Horiba Jobin Yvon, Labram HR) using a 632.8 nm laser. An average of 3 points per sample was collected and normalized to the D-band intensity. The areas were obtained from mathematical deconvolution using the software OriginPro 2019 ®.

The morphology of MC particles, the sulfur distribution in the S/C composite (MC-S), and the chemical composition of the screen-printed gold electrodes modified with thiolated mesoporous carbon (SPAuE/MC-SH) were analyzed by field emission scanning electron microscopy (FE-SEM) without coating on an Apreo 2 SEM microscope, with an accelerating voltage of 5 kV, and equipped with an energy-dispersive X-ray spectroscopy (EDX) analyzer. Fourier-transformed infrared (FTIR) spectroscopy was used to identify the primary functional groups present in the carbonaceous material before and after sulfur infiltration. Spectra were acquired using a Thermo Fisher Scientific Nicolet 6700 FTIR spectrophotometer in transmittance mode with 32 scans over a 4000-800 cm^-1^ range. Baseline correction and smoothing were performed using Ominic software (Thermo Fisher Scientific Inc, MA, USA). The surface elemental composition of MC, MC-S, and SPAuE/C-SH was determined by X-ray photoelectron spectroscopy (XPS) using a Specs NAP-XPS spectrometer equipped with a PHOIBOS 150 1D-DLD analyzer. It used monochromatic Al Kα (1486.7 eV, 13 kV, 100 W) light and an energy step of 85.36 eV for the survey spectrum. The energy step for high-resolution spectra was 20 eV for each element. The step values were 1 and 0.1 eV for the survey and high-resolution spectra, respectively. The spectrum was analyzed using CasaXPS software.

The electrochemical measurements were performed in a potentiostat/galvanostat MultiPalmSens 4 with MultiTrace software version 4.5. SPAuEs were purchased from Metrohm DropSens (ref. DRP-220-BT). The electrodes consist of a three-electrode cell configuration with a working gold electrode (4 mm diameter), a gold counter electrode, and a silver (Ag) pseudo-reference electrode, all printed on a ceramic substrate. All potentials are reported relative to a silver pseudo-reference electrode.

**3. Sulfur-doped mesoporous carbon preparation**

The mesoporous carbon CMK-3 (MC) was synthesized with SBA-15 as a template to replicate its morphology and porosity. The SBA-15 silica was grafted with aluminum to create acidic sites for the polymerization of furfuryl alcohol, which served as a carbon source. For this purpose, the silica was dispersed in an ethanolic AlCl_3_ solution (Si/Al = 1.75) for 1 h at room temperature. Vacuum filtration was used to recover the powder, which was then dried for 12 h at 80 °C. A second dispersion was developed for 3 h. The resultant powder was calcined in static air at 550 °C for 3 h (1 °C min^-1^). The powder was impregnated with a volume of furfuryl alcohol up to 40% greater than the pore volume (Table S1 shows the pore volume obtained for SBA-15), then thermally treated at 35 °C for 1 h, 100 °C for 5 h, and 160 °C for 5 h. A second impregnation with furfuryl alcohol was performed using 60% of the initial volume, followed by the previously described heat activation. Finally, it was carbonized in a nitrogen atmosphere at 850 °C, ramping at 3 °C min^-1^, and held for 5 h. The silica template was removed using 50 mL of a 10% v/v HF solution [1]. As described in previous reports, sulfur infiltration was performed using the melting-diffusion technique. MC and sulfur were macerated for 15 min to generate the composite (MC-S) with a 70:30 wt.% ratio (S/C); to obtain 1 g of the MC-S composite, 0.7 g of MC and 0.3 g of elemental sulfur were used. Then, it was suspended in carbon disulfide and magnetically agitated for 30 min, after which the solvent was removed by evaporation. The mixture was heated to 155 °C in a Teflon autoclave container and held for 6 h to introduce sulfur into the mesoporous carbon's pores [2].

**Table S1.** Textural properties of the silica template (SBA-15), mesoporous carbon CMK-3-type (MC), and sulfur/carbon composite (MC-S).

| ***Sample*** |  | ***Textural Properties*** | | | |
| --- | --- | --- | --- | --- | --- |
|  | ***S_BET_ (m^2^g^-1^)*** | | ***V_T_ (cm^3^ g^-1^)*** | ***Dp (nm)*** | ***A_D1_/A_G_*** |
| SBA-15 | 532.8 | | 1.3 | 9.6 | ---- |
| MC | 1462.2 | | 1.7 | 4.7 | 4.3 |
| MC-S | 29.95 | | 0.1 | ---- | 4.5 |


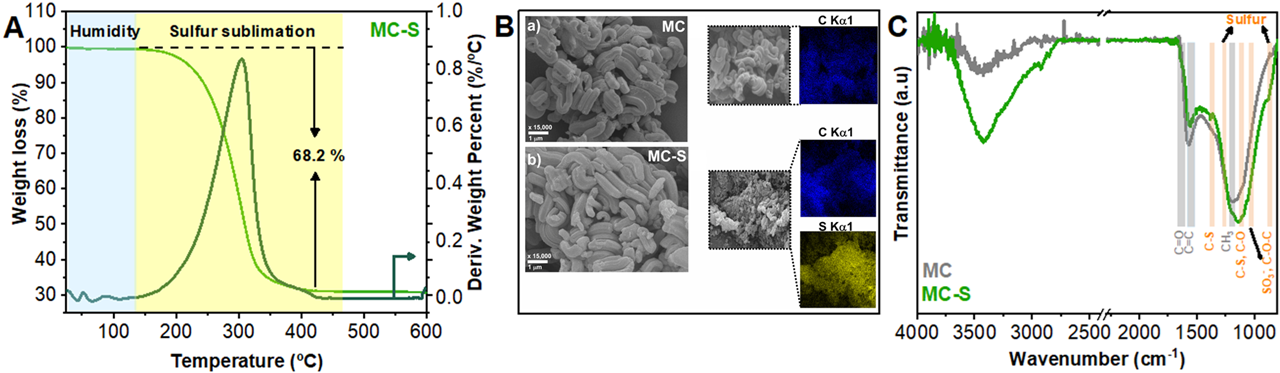


***Figure S1.*** *A) Thermogravimetric analysis of the MC-S composite, B) SEM-EDX micrographs for the carbonaceous material before and after sulfur incorporation, and C) Infrared spectroscopy of the materials studied.*


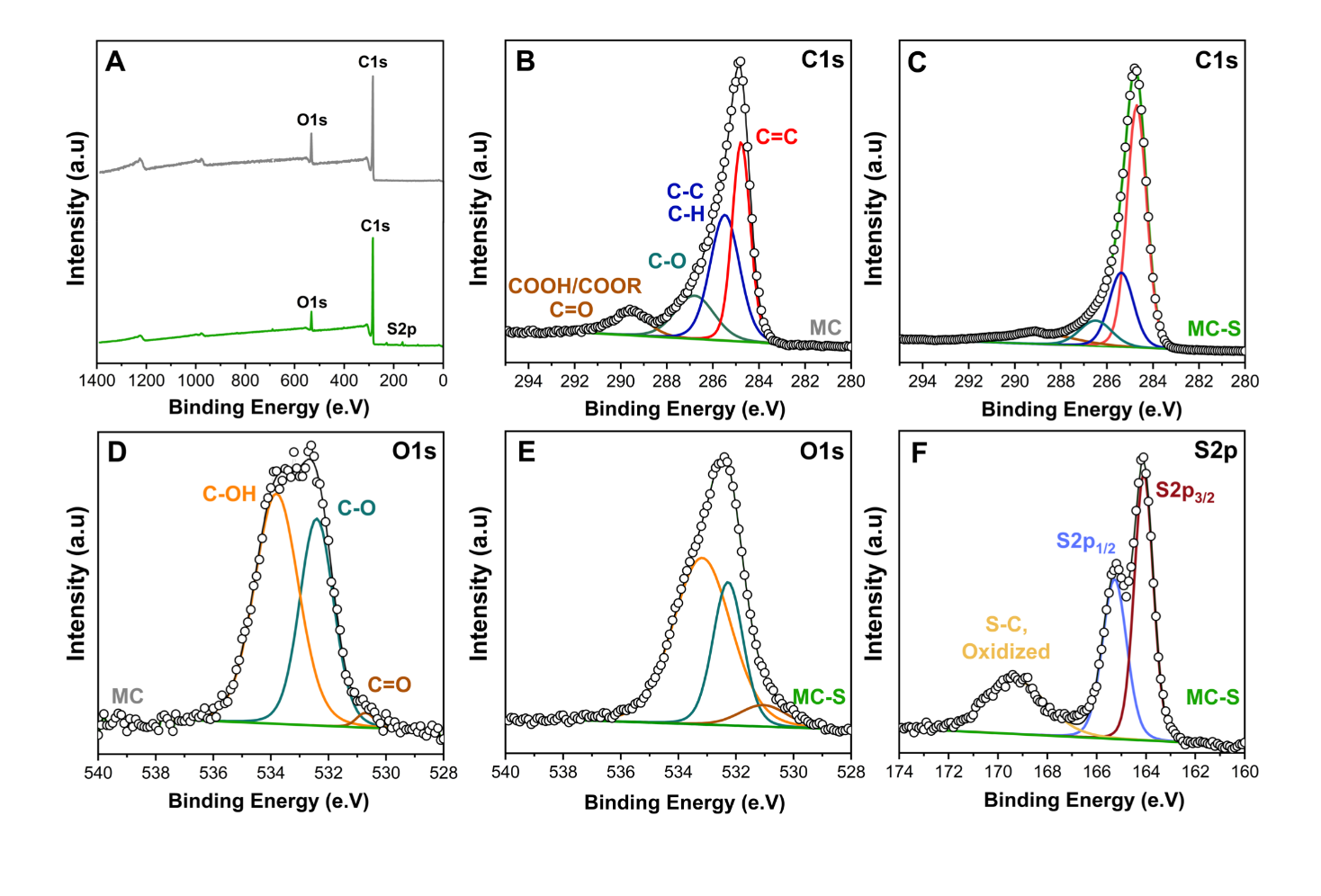


***Figure S2.*** *A) XPS survey spectrum for MC and MCS, B-C) high-resolution spectrum for C1s, D-E) high-resolution spectrum for O1s, and F) high-resolution spectrum for S2p.*

# **4. MC-S functionalization with thiol groups**

The thiol functionalization of the MC-S composite was done following a reported procedure [3]. Briefly, the MC-S composite was dispersed at a concentration of 1 mg mL^-1^ in 10 mL of ethanol and sonicated for 15 min. It was then irradiated with a UV lamp (λ = 254 nm, power 6.0 W) at a distance of 10 cm and magnetically stirred for 30 min. Sulfur radicals generated by UV irradiation were reduced by adding 12 mg of NaBH_3_CN, yielding a final concentration of 1.2 mg mL^-1^. The mixture was sonicated for 15 min and magnetically stirred for 60 min at room temperature (25°C).

# **5. SPAuE pretreatment**

First, the SPAuE surface was activated by cyclic voltammetry (CV) in 0.1 M H_2_SO_4_ in a potential window between +1.6 V and 0 V at a scan rate of 0.1 V s^-1^ for 5 cycles (**Figure S3A**). When the SPAuE surface was activated, the real surface area (*A_real_*) and surface roughness (*R*) were calculated using **Equations 1** and **2**, respectively. To ensure reproducibility in each SPAuE, *R* was maintained at 10.2 ± 0.3. The electroactive area (*A_e_*) was calculated using the Randles-Sevcik equation from CV experiments (**Figure S3B**) conducted in a PBS solution containing 5 mM [Fe(CN)_6_]^4-/3-^ at a scan rate of 0.05 V s^-1^, to verify the proper SPAuE surface activation (**Equation 3**). The *A_e_* was calculated to be 18 ± 0.4 mm^2^, indicating successful activation.


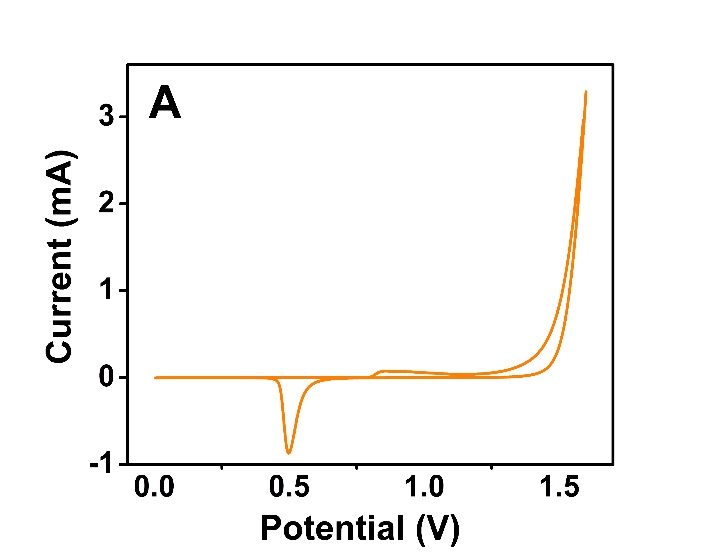

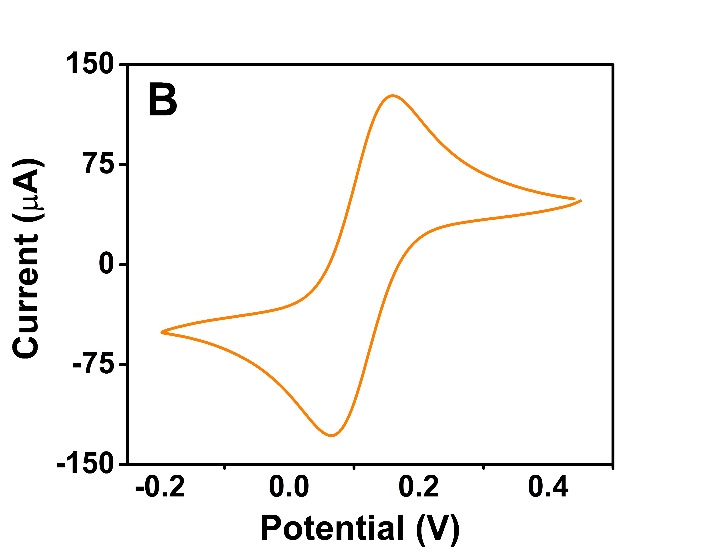


***Figure S3****.* ***A****)* *Cyclic voltammogram of SPAuE in 0.1 M of H_2_SO_4_ aqueous solution at a sweep rate of 0.1 V s^-1^. A_real_ was calculated by integrating the gold oxide reduction peak.* ***B****) Cyclic voltammogram in 5 mM [Fe(CN)_6_]^4-/3-^ / 10 mM PBS. A_e_ was calculated using the Randles-Sevcik equation.*

$A_{real}= \frac{Q_{AuO}}{\rho}$ Eq. (1)

*Q_AuO_* is the reduction charge, which was calculated by integrating the cathodic current profile area of the 5^th^ CV cycle (526.2 ± 14.2 μC). 𝜌 = charge density consumed on the gold surface (theoretical value is 410 μC cm^-2^) [4]. *A_geo_* is the geometric area of the working electrode reported by the manufacturer (0.126 cm^2^).

$R= \frac{A_{geo}}{A_{real}}$ Eq. (2)

$I_{p}=\pm0.446nFA_{e}C\sqrt{\frac{nFDv}{RT}}$ Eq. (3)

Where *I_p_* is the current intensity, *n* is the number of electrons involved in the electrochemical reaction (n = 1), *C* and *D* are the redox probe concentration (5 mM) and diffusion coefficient (7.62 x 10^-6^ cm^2^ s^-1^), respectively, *F* is the Faraday's constant (96485 C mol^-1^), ν is the scan rate (0.05 V s^-1^), *R* is the universal gas constant (8.314 J K^-1^ mol^-1^), and *T* is the absolute temperature (T = 298.15 K).

# **6. Immunosensor preparation**

The SPAuE modification with thiolated-MC (MC-SH) was performed by chemisorption of sulfhydryl groups onto the gold surface. It was achieved by drop-casting 1 μL onto the working electrode surface in ten steps at 25°C, totaling 10 μL [5]. This modification also generated thiol groups on the SPAuE surface, which reacted with the maleimide of MPA, thereby functionalizing the SPAuE with carboxylic acids. 12 µL of 1.5 % MPA (w/v) in PB pH 6.5 was incubated on the modified SPAuE/MC-SH for 20 h at 4 °C [6]. Each modified interface was washed with distilled water and air-dried at room temperature.

Next, the MPA carboxylic acids were activated by adding 12 µL of EDC/NHS (8/2 mM) dissolved in 25 mM MES buffer, pH 5.5, on the working electrode. The activation reaction was performed for 1 min, and the modified SPAuE was washed with distilled water [7]. The biorecognition element was immobilized by covalent coupling to the SPAuE/C-SH/MPA interface, with 12 µL of 100 µg mL^-1^ anti-CEA antibody solution incubated for 90 min at 25°C in a dark, humid chamber. Next, 12 µL of 1.0 % BSA (wt/v) was added to the biofunctional interface for 30 min at 25 °C to block unreacted N-hydroxysuccinimide esters. Molecular target detection was performed by adding 12 µL of CEA at known concentrations dissolved in PBS 1X, pH 7.4, to the immunosensor, and the antigen-antibody reaction was carried out for 60 min at 25°C in a dark, humid chamber. After each bioconjugation step, the biofunctional interfaces were washed with distilled water and air-dried at 25°C.

# **7. Electrochemical measurements**

CV, electrochemical impedance spectroscopy (EIS), and differential pulse voltammetry (DPV) were performed using the 5 mM [Fe(CN)_6_]^4-/3-^ redox probe in PBS 1X, pH 7.4, as the supporting electrolyte. The potential window for CV was scanned from +0.45 V to −0.2 V at 0.05 V s^-1^ for 3 cycles. EIS was conducted at the formal potential of the redox probe (E_dc_ = 0.115 V), the amplitude of the sinusoidal wave was E_ac_ = 10 mV, and frequencies ranged from 100 kHz to 0.1 Hz. Impedance spectra were analyzed by fitting an equivalent electrical circuit in MultiTrace using the Levenberg–Marquardt algorithm. DPV measurements were recorded in a potential range between − 0.4 and + 0.4 V at 0.05 V s^−1^ with a pulse amplitude of 0.05 V and a pulse width of 0.025 s.

# **8. Analytical performance**

The analytical performance of the immunosensor was evaluated by detecting different concentrations of the CEA glycoprotein (1, 2.5, 5, 7.5, and 10 ng mL^-1^). The molecular biorecognition event was monitored by the normalized change in DPV current intensity, calculated as $the relative response RR \%=(\Delta I/I_{0})=[(I_{Immunosensor} - I_{CEA})/I_{Immunosensor}] x 100 \%$, where $I_{Immunosensor}$ represents the DPV current intensity of the BSA/anti-CEA/MPA/MC-SH/SPAuE biofunctional interface and $I_{CEA}$ represents the DPV current intensity after binding to the CEA glycoprotein. The limits of detection (LOD) and quantification (LOQ) of the immunosensor were determined by using the 3-sigma and 10-sigma criteria, respectively, with *LOD* = *3S_b_/m* and *LOQ* = *10S_b_/m*, where *S_b_* is the standard deviation for the normalized change in DPV current intensity of the blank, and *m* is the slope of the calibration curve.

# **9. Analysis of human serum samples**

The Colombian Cancer Foundation, Clinica Vida, from Medellín, Colombia, collected 1-2 ml of six random blood samples from four patients diagnosed with CRC and two healthy individuals between November 2022 and April 2023, and sent them to the Max Planck Tandem Group, keeping the cold chain and stored at -20 ºC for further evaluation using the biosensor. The population tested included males and females aged 18 or older, and the results were obtained in accordance with established ethical and biosafety standards. The study was approved by the Fundación Colombiana de Cancerología Clínica Vida Ethical Committee #121 on February 22, 2023. The samples were diluted in PBS buffer and analyzed with the immunosensor by adding 12 µL to the working electrode for 60 min at 25°C. The concentration of CEA in the serum samples was estimated based on the relationship between current intensity and concentration. The assay results were compared with those obtained using a commercial CEA ELISA kit.

**Table S2.** Elemental composition of the electrode surface by EDX analysis (atomic %).

| **Element** | **Atomic (%)** | **Error (%)** |
| --- | --- | --- |
| Au | 49.2 | 0.2 |
| C | 47.7 | 0.3 |
| O | 0.4 | 0.3 |
| S | 2.6 | 0.4 |


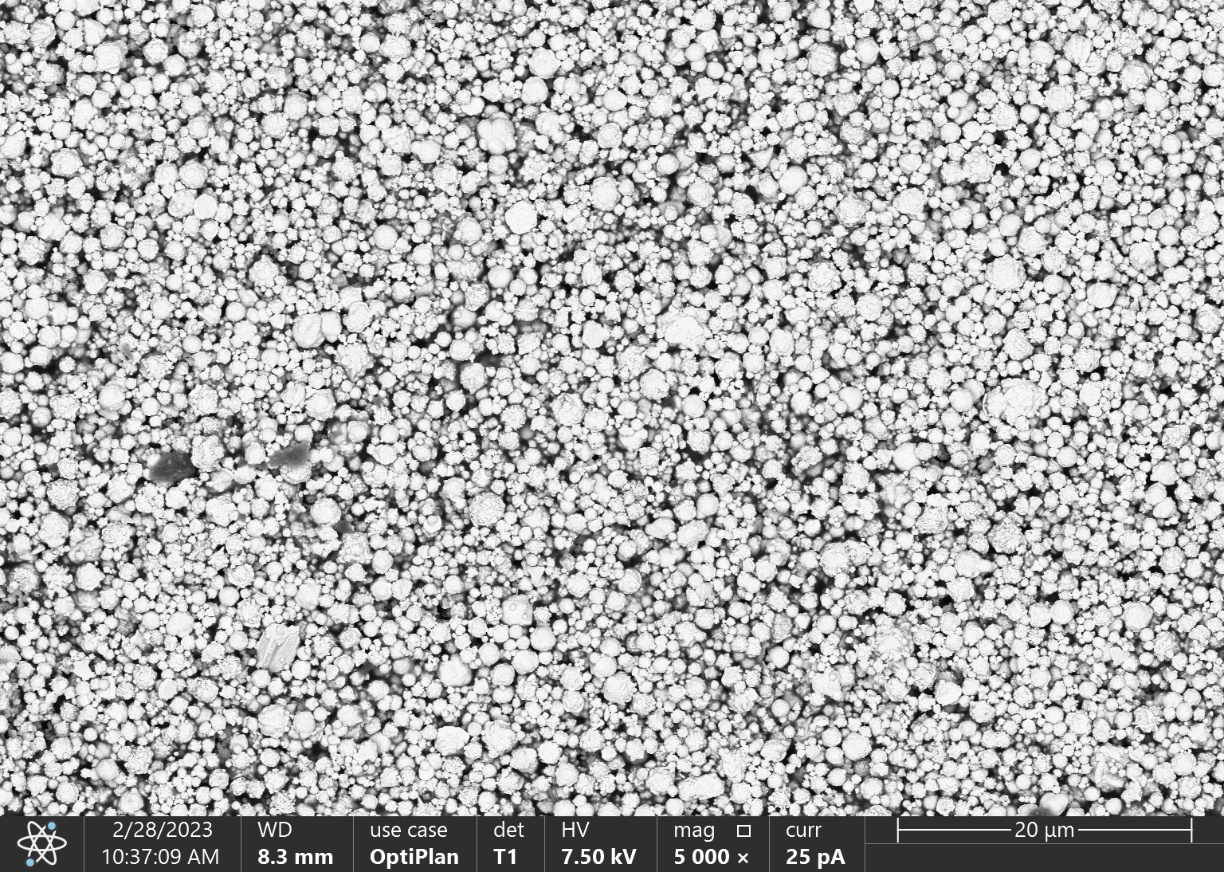


**A**


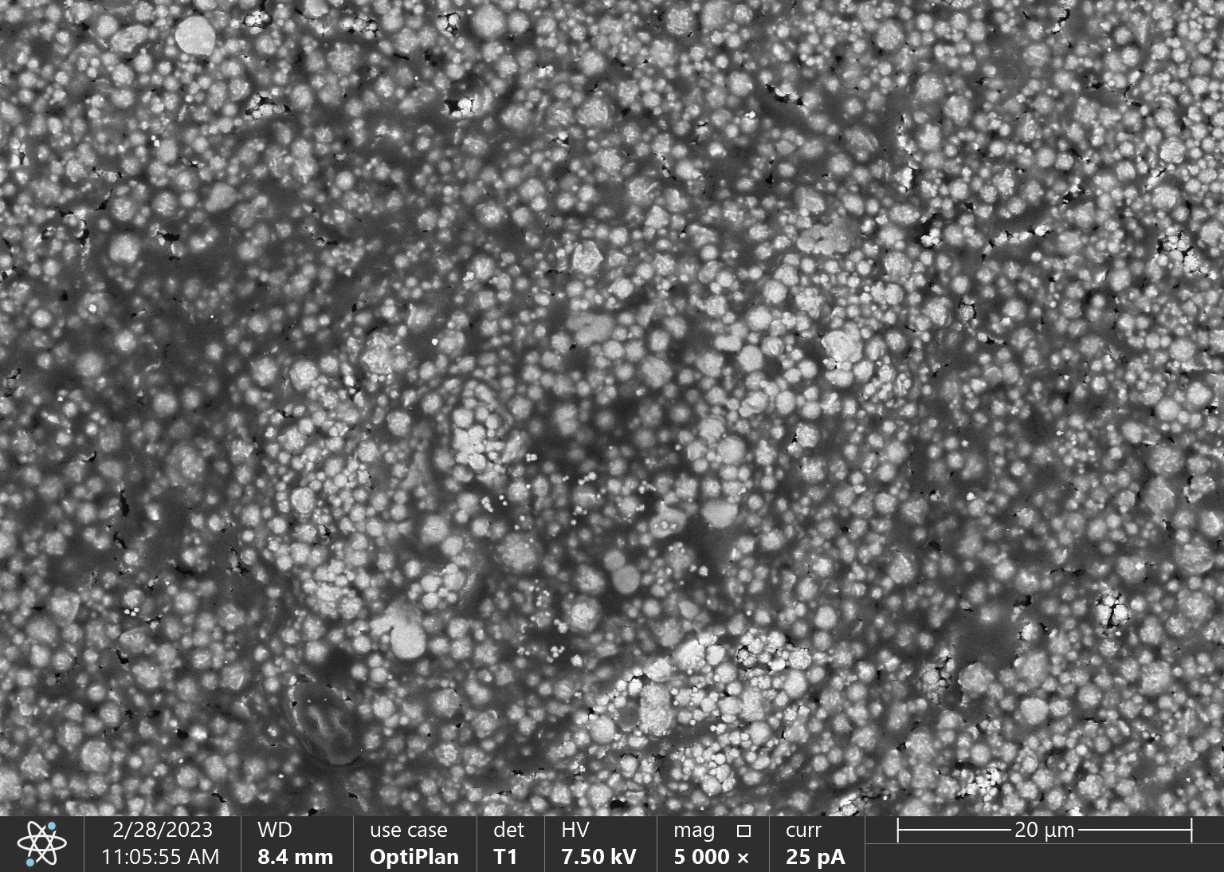


**B**


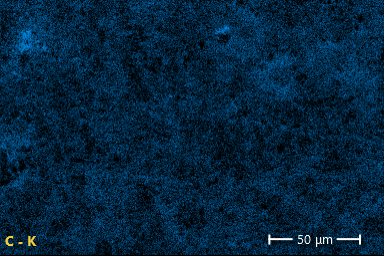


**C**


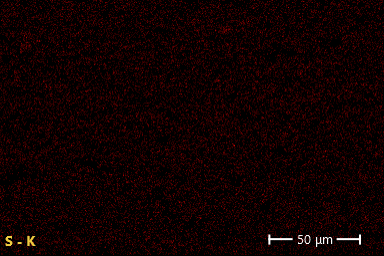


**D**

*Figure S4. FE-SEM micrograph of the electrodes. A) SPAuE bare. B) MC-SH/SPAuE modified electrode. EDS mapping of C ) carbon and D) sulfur elements, respectively.*

**Table. S3.** Data from the electrochemical characterization of the SPAuEs modified with MC-SH, MPA/MC-SH/SPAuE, EDC/NHS/MPA/MC-SH/SPAuE, anti-CEA/MPA/MC-SH/SPAuE, BSA/anti-CEA/MPA/MC-SH/SPAuE, and CEA/BSA/anti-CEA/MPA/MC-SH/SPAuE interfaces by CV and EIS. The electroactive area for each modification step was calculated from the CV using the 5 mM [Fe(CN)_6_]^4−/3−^ redox probe / PBS 1X, pH 7.4 at a scan rate of 50 mV s^-1^: anodic electroactive area (*A_a_*), cathodic electroactive area (*A_c_*), average electroactive area (*A_e_*), anodic and cathodic peak separation (*∆Ep*). Data from EIS experiments: Charge-transfer resistance (*R_ct_*), electrolytic solution resistance (*R_s_*), the Warburg impedance (*Z_w_*), constant phase element (*CPE*) with pre-exponential factor (*P*), and exponent (*n*).

| **Interface** | ***A_a_***  **(mm^2^)** | ***A_c_***  **(mm^2^)** | ***A_e_***  **(mm^2^)** | ***∆Ep***  **(mV)** | ***R_ct_***  **(Ω)** | ***R_S_***  **(Ω)** | ***Z_w_***  **(Ω s^0.5^)** | ***CPE*** | |
| --- | --- | --- | --- | --- | --- | --- | --- | --- | --- |
|  |  |  |  |  |  |  |  | ***P* (µF s^n-1^)** | ***n*** |
| **SPAuE** | 18.2 ± 0.2 | 17.7 ± 0.2 | 18.0 | 89 | 15.8 ± 2.3 | 28.1 ± 2.7 | 248.9 | 49.6 | 0.80 |
| **MC-SH** | 5.3 ± 0.8 | 5.7 ± 0.8 | 5.5 | 210 | 1198 ± 80 | 27.1 ± 0.1 | 481.0 | 13.3 | 0.77 |
| **MPA** | 3.2 ± 0.5 | 2.5 ± 0.4 | 2.9 | 323 | 4392 ± 316 | 29.3 ± 1.9 | 397.3 | 6.0 | 0.81 |
| **EDC/NHS** | 15.2 ± 0.4 | 14.7 ± 0.2 | 15.0 | 97 | 86.7 ± 7.4 | 26.3 ± 5.2 | 518.1 | 5.7 | 0.78 |
| **Anti-CEA** | 14.1 ± 0.5 | 13.7 ± 0.5 | 13.9 | 100 | 143.2 ± 8.9 | 30.8 ± 4.6 | 474.0 | 6.0 | 0.80 |
| **BSA** | 13.7 ± 0.5 | 13.6 ± 0.2 | 13.6 | 107 | 184.3 ± 29.8 | 29.6 ± 1.3 | 344.0 | 4.9 | 0.82 |
| **CEA** | 12.4 ± 0.2 | 13.0 ± 0.7 | 12.7 | 127 | 324.8 ± 27.1 | 28.0 ± 1.2 | 500.0 | 8.5 | 0.80 |


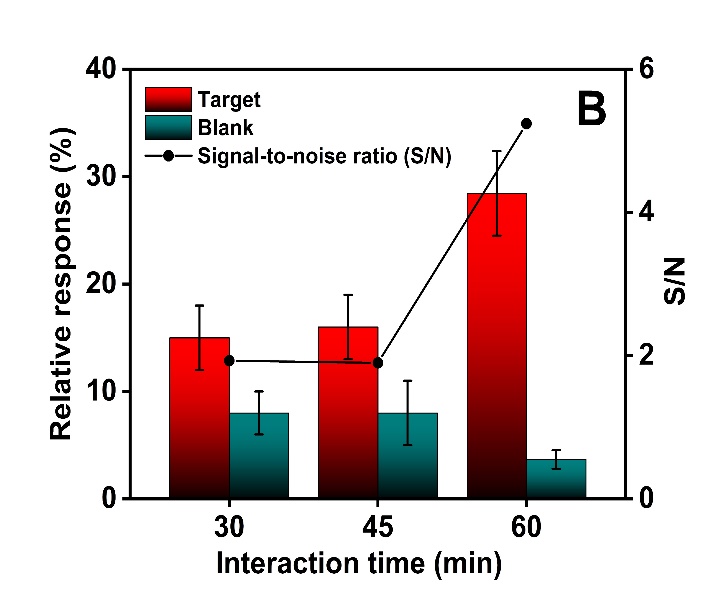

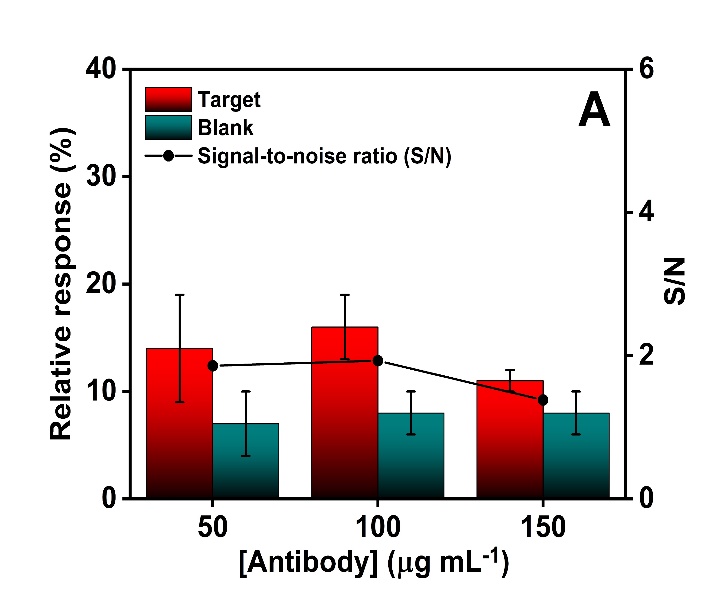


***Figure S5****. Optimized experimental parameters related to the antibody bioreceptor using 10 ng mL^-1^ of CEA glycoprotein.* ***A****) Optimal anti-CEA antibody concentration (50, 100, and 150 µg mL^-1^).* ***B****) Optimal antigen-antibody interaction time (30, 45, and 60 min).*


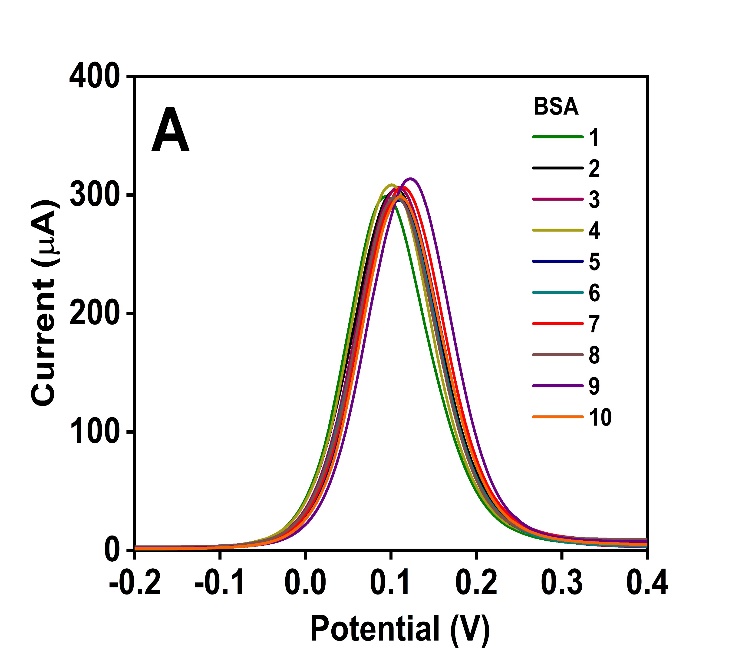

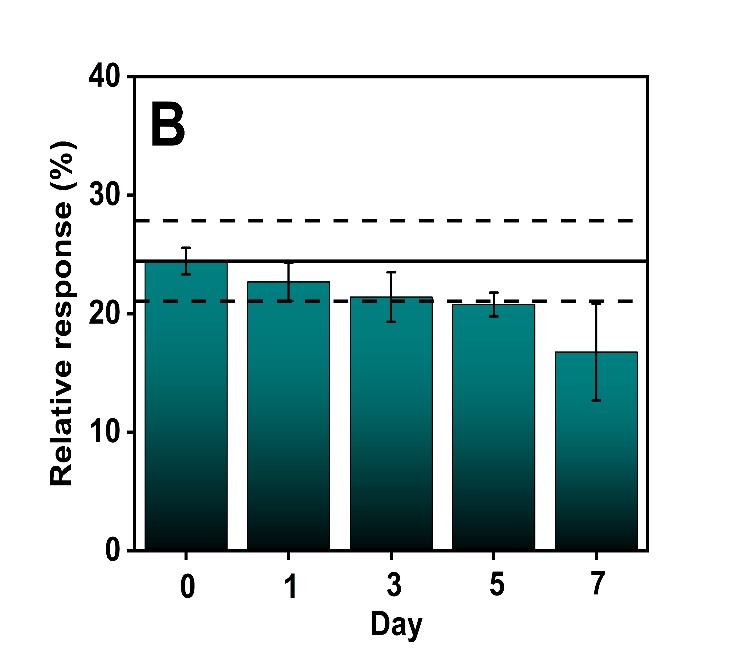


***Figure S6****. Immunosensor assembly reproducibility and device stability.* ***A****) DPV current response for ten replicates of immunosensors assembled up to the blocking step with BSA on independent electrodes.* ***B****) Time stability study of the immunosensor to detect 10 ng mL^−1^ of CEA glycoprotein.*

**Table S4.** Assay results of human serum samples with the standard ELISA method and the electrochemical immunosensor.

| **Sample No. ^a^** | **ELISA method ^b^**  **(ng mL^-1^)** | **RSD**  **(%, n=2)** | **Immunosensor ^b^**  **(ng mL^-1^)** | **RSD**  **(%, n=2)** |
| --- | --- | --- | --- | --- |
| 1 | 0.57 ± 0.02 | 2.6 | N. D | - |
| 2 | 1.05 ± 0.06 | 6.0 | N. D | - |
| 3 | 6.66 ± 0.91 | 14.4 | 4.86 ± 0.06 | 1.3 |
| 4 | 7.30 ± 0.70 | 9.6 | 8.16 ± 0.63 | 7.7 |
| 5 | 8.47 ± 0.66 | 7.8 | 7.33 ± 0.41 | 5.6 |
| 6 | 10.07 ± 0.02 | 0.2 | 11.26 ± 0.38 | 3.4 |

^a^Samples 1-2 are the serum samples from healthy people, and samples 3-6 are the serum samples from cancer patients.

^b^Data are the mean ± standard deviation of two measurements.

N. D Non-detectable. CEA concentration was lower than the LOQ of the immunosensor.

**Table S5.** Label-free electrochemical immunosensors for the detection of CEA based on screen-printed electrodes

| **Sensing platform** | **Detection technique** | **Limit of detection**  **(ng mL^-1^)** | **Linear range**  **(ng mL^-1^)** | **Assay time***  **(min)** | **Matrix** | **Analytical relevance** | **Ref.** |
| --- | --- | --- | --- | --- | --- | --- | --- |
| **SPCE/Au-MoO_3_-Chitosan** | LSV | 5.0 × 10^-4^ | 0.001 – 1.0 | 30 | Clinical serum samples | Validated in clinical serum; linear range limited to low concentration levels | [8] |
| **SPCE/Nitrogen-rich mesoporous carbon** | SWV | 9.0 × 10^-3^ | 0.009 – 1000 | 15 | Spiked serum | Demonstrated only in spiked serum; clinical applicability not validated | [9] |
| **SPCE/Cu-Ni MOF** | DPV | 1.6 × 10^-4^ | 0.001 – 500 | 120 | Clinical serum samples | Applicable to clinical samples but requires a long assay time | [10] |
| **SPCE/3D porous chitosan cryogel-AuNPs-PEDOT-PB** | DPV | 5.1 × 10^-8^ | 1.0 × 10^-7^ – 1.0 | 15 | Spiked serum | Ultra-low LOD but evaluated only in spiked serum; limited clinical validation | [11] |
| **SPCE/Graphene-Thionine-AuNPs** | DPV | 1.0 × 10^-2^ | 0.05 -500 | - | Clinical serum samples | Tested in clinical samples; assay time not reported | [12] |
| **SPCE/Graphene nanoplatelets-MnO_2_/Fe_3_O_4_@Au** | LSV | 1.0 × 10^-4^ | 0.001 – 100 | 120 | Clinical serum samples |  | [13] |
|  | EIS | 3.0 × 10^-4^ |  |  |  | Tested in real samples. Long assay time, complex nanoarchitecture |  |
| **SPAuE/Polyoctopamine** | EIS | 1.9 × 10^-3^ | 0.02 – 180 | 20 | Spiked serum | \|  \| \| --- \|   Demonstrated only in spiked serum; limited relevance for real clinical samples | [14] |
| **SPCE/** **Poly-(pyrrole-3-carboxylic acid)** | EIS | 3.3 × 10^-2^ | 0.10 - 100 | 60 | Spiked serum | \|  \| \| --- \|   Demonstrated only in spiked serum | [15] |
| **SPAuE/built-in thiol mesoporous carbon/MPA** | DPV | 1.0 | 1.0 - 10 | 60 | Clinical serum samples | Linear range matches clinically relevant concentrations; detection in clinical samples | This work |

|  |
| --- |

*Incubation time of antigen-antibody interaction. MOF: metallic organic framework. MPA: 3-maleimidepropionic acid. PB: Prussian blue. PEDOT: Poly(3,4-ethylenedioxythiophene).

# **References**

1. López M, Palacio R, Mamede AS, et al (2020) Hydrodeoxygenation of guaiacol into cyclohexane over mesoporous silica supported Ni–ZrO2 catalyst. Microporous and Mesoporous Materials 309:. https://doi.org/10.1016/j.micromeso.2020.110452

2. Laverde J, Rosero-Navarro NC, Miura A, et al (2022) Impact of Sulfur Infiltration Time and Its Content in an N-doped Mesoporous Carbon for Application in Li-S Batteries. Batteries 8:. https://doi.org/10.3390/batteries8060058

3. Mao J, Wang Y, Zhu J, et al (2018) Thiol functionalized carbon nanotubes: Synthesis by sulfur chemistry and their multi-purpose applications. Appl Surf Sci 447:235–243. https://doi.org/10.1016/j.apsusc.2018.03.188

4. Garrote BL, Santos A, Bueno PR (2020) Label-free capacitive assaying of biomarkers for molecular diagnostics. Nature Protocols 2020 15:12 15:3879–3893. https://doi.org/10.1038/s41596-020-0390-9

5. Rojas D, Della Pelle F, Del Carlo M, et al (2019) Nanohybrid carbon black-molybdenum disulfide transducers for preconcentration-free voltammetric detection of the olive oil o-diphenols hydroxytyrosol and oleuropein. Microchimica Acta 186:. https://doi.org/10.1007/s00604-019-3418-5

6. Hermanson GT (2013) Modification of Sulfhydryls with BMPA. In: Audet J, Preap M (eds) Bioconjugate Techniques, Third. pp 193–194

7. Cruz-Pacheco AF, Quinchia J, Orozco J (2022) Cerium oxide–doped PEDOT nanocomposite for label-free electrochemical immunosensing of anti-p53 autoantibodies. Microchimica Acta 2022 189:6 189:1–13. https://doi.org/10.1007/S00604-022-05322-5

8. Cotchim S, Kongkaew S, Thavarungkul P, et al (2024) A dual-electrode label-free immunosensor based on in situ prepared Au–MoO3-Chi/porous graphene nanoparticles for point-of-care detection of cholangiocarcinoma. Talanta 272:. https://doi.org/10.1016/j.talanta.2024.125755

9. Mehta D, Kaur S, Nagaiah TC (2024) Realizing the label-free sensitive detection of carcinoembryogenic antigen (CEA) in blood serum via a MNC-decorated flexible immunosensor. Analytical Methods 16:1473–1479. https://doi.org/10.1039/d3ay02073h

10. Shu Y, Yan L, Ye M, et al (2023) A bimetallic metal-organic framework with high enzyme-mimicking activity for an integrated electrochemical immunoassay of carcinoembryonic antigen. Analyst 148:4721–4729. https://doi.org/10.1039/d3an01221b

11. Choosang J, Khumngern S, Nontipichet N, et al (2023) 3D porous CS-AuNPs-PEDOT-PB nanocomposite cryogel for highly sensitive label-free electrochemical immunosensor for carcinoembryonic antigen determination. Microchemical Journal 187:. https://doi.org/10.1016/j.microc.2023.108435

12. Wang Y, Xu H, Luo J, et al (2016) A novel label-free microfluidic paper-based immunosensor for highly sensitive electrochemical detection of carcinoembryonic antigen. Biosens Bioelectron 83:319–326. https://doi.org/10.1016/j.bios.2016.04.062

13. Butmee P, Tumcharern G, Thouand G, et al (2020) An ultrasensitive immunosensor based on manganese dioxide-graphene nanoplatelets and core shell Fe3O4@Au nanoparticles for label-free detection of carcinoembryonic antigen. Bioelectrochemistry 132:. https://doi.org/10.1016/j.bioelechem.2019.107452

14. Shamsuddin SH, Gibson TD, Tomlinson DC, et al (2021) Reagentless Affimer- and antibody-based impedimetric biosensors for CEA-detection using a novel non-conducting polymer. Biosens Bioelectron 178:. https://doi.org/10.1016/j.bios.2021.113013

15. Iordănescu A, Tertis M, Cernat A, et al (2018) Poly-(pyrrole-3-carboxylic acid) Based Nanostructured Platform for the Detection of Carcinoembryonic Antigen. Electroanalysis 30:1100–1106. https://doi.org/10.1002/elan.201700803
